# When are Two‐Dimensional Covalent Organic Frameworks Good Electrodes for Lithium Ion Batteries? A Data‐Driven Analysis

**DOI:** 10.1002/advs.78599

**Published:** 2026-11-01

**Authors:** Preeti Bhauriyal, Shubhajit Das, Thomas Heine

**Affiliations:** ^1^ Faculty of Chemistry and Food Chemistry Technische Universität Dresden Dresden Germany; ^2^ Department of Chemistry & IBS of Nanomedicine Yonsei University Seoul Republic of Korea; ^3^ Department of Chemistry Ewha Womans University Seoul Republic of Korea

**Keywords:** all‐organic Li battery, anode‐cathode pairs, CoRE COF, covalent organic frameworks, density functional theory, high‐throughput screening, lithium ion battery, machine learning

## Abstract

Two‐dimensional covalent organic frameworks (2D COFs) are emerging as sustainable organic electrode candidates for lithium‐ion batteries. Despite more than a decade of research, existing heuristics for designing electroactive COFs lack generality, and the suitability of these materials as alternatives to conventional electrodes remains poorly resolved. Here, we map structure–electrode–function relationships across 1099 experimentally synthesized 2D COFs from the CoRE COF database by developing a data‐driven framework integrating density‐functional theory computations with machine learning models and establishing atomic‐level design rules governing electrode behavior. We uncover that cathode‐like character originates from dense, multidentate nitrogen and oxygen rich coordination pockets at pore walls, characteristic of imide, acylhydrazones, keto–amine linkages, and π‐macrocycles, whereas anode‐like behavior is encoded by spatially separated redox sites and π‐rich interlayer galleries that sustain weak, reversible Li^+^ intercalation. Sequential voltage, capacity, and conductivity‐based screening across the database yields 14 anode and 14 cathode candidates. We show that the known chemical space of 2D COFs cannot outperform inorganic electrodes in terms of operating voltages. However, strategic pairing of 2D anode|cathode pairs within an all‐organic lithium battery architecture delivers full‐cell voltages exceeding 2 V with capacities above 200 mAh g^−1^ on both electrodes, surpassing small organic molecules and polymer counterparts.

## Graphical abstract

A data‐driven framework integrating density‐functional theory with machine learning models screens two‐dimensional covalent organic frameworks (2D COFs) from CoRE COF database to reveal atomic‐level design rules: N/O‐rich coordination pockets drive cathode behavior, while separated redox sites and π‐rich galleries enable anode function in Li‐ion batteries. Strategic pairing of 2D COFs as anode|cathode has the potential for an all‐organic lithium battery architecture.

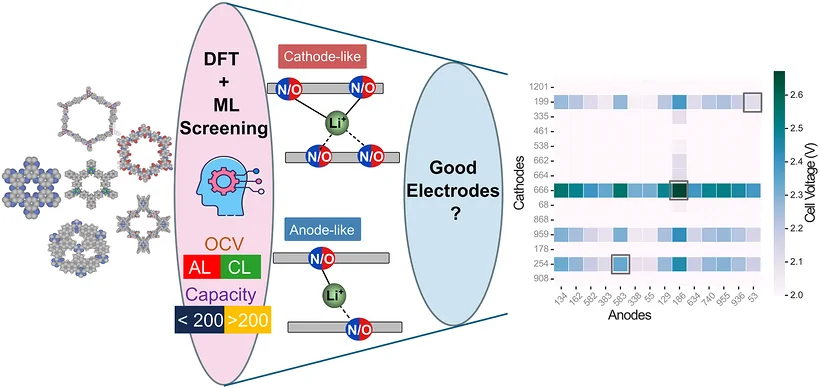

## 1 Introduction

Sustainable organic electrodes have gained significant attention as promising alternatives to conventional inorganic electrodes, some of which suffer from critical limitations including resource scarcity, high cost, high energy consumption, and environmental concerns [1, 2, 3]. Two dimensional covalent organic frameworks (2D COFs) have been studied in terms of their suitability as organic electrodes for batteries [4, 5, 6, 7, 8, 9, 10, 11]. 2D COFs are stacks made of ordered porous polymers composed of lightweight elements linked through strong covalent bonds, featuring reticular design, high surface area, and abundantly accessible pore channels. Their structural tunability allows the deliberate incorporation of redox‐active functionalities (RAFs) and the tailoring of pore environments to facilitate ion adsorption and transport [12, 13, 14]. Meanwhile, with favorable stacking, conjugation, and/or conductive additives, COFs have shown improved electron conductivity and cycling stability [15, 16, 17, 18]. Despite extensive research efforts over the past decade, design of electroactive COFs remains largely empirical because the structure‐performance relationships governing their electrode behavior are poorly understood. As a result, their viability as replacements for conventional anode and cathode materials in lithium‐ion batteries (LIBs), or as both electrodes in all‐organic Li batteries (AOLBs) remains unclear.

A suitable metal ion battery (MIB) electrode material shall deliver an appropriate operating voltage and reversible capacity, enable sufficiently fast ion and electron transport (intrinsically or through electrode engineering), and maintain electrochemical, thermal, and mechanical stability over repeated cycling. Among these general performance metrics, the primary thermodynamic descriptor that differentiates potential anodic and cathodic behavior is the redox potential referenced to the metal electrode (voltage vs M/M^+^ in V). In practical terms, materials operating at lower voltage vs. M/M^+^ are more reducing and are therefore typically deployed as anodes, whereas the ones with higher voltage are more oxidizing and positioned as cathodes. Since COFs lack well‐defined working voltage windows, there is a range where both anode and cathode behavior might be feasible. Based on our focused survey of experimentally reported 2D COF electrodes [19, 20, 21, 22, 23, 24, 25, 26, 27, 28, 29, 30, 31, 32, 33, 34, 35, 36], we identify ∼1.6 V vs Li/Li^+^ as a reasonable classification threshold, where COFs with a minimum voltage of 1.6 V or greater can be projected as cathodes and COFs with a voltage of less than 1.6 V as anodes for LIBs (Figure 1; Figure S1 and Table S1). This is fully consistent with the reduction potential window of the redox‐functionalities frequently observed in COFs as previously schemed by Haldar et al. [37].

**FIGURE 1:**
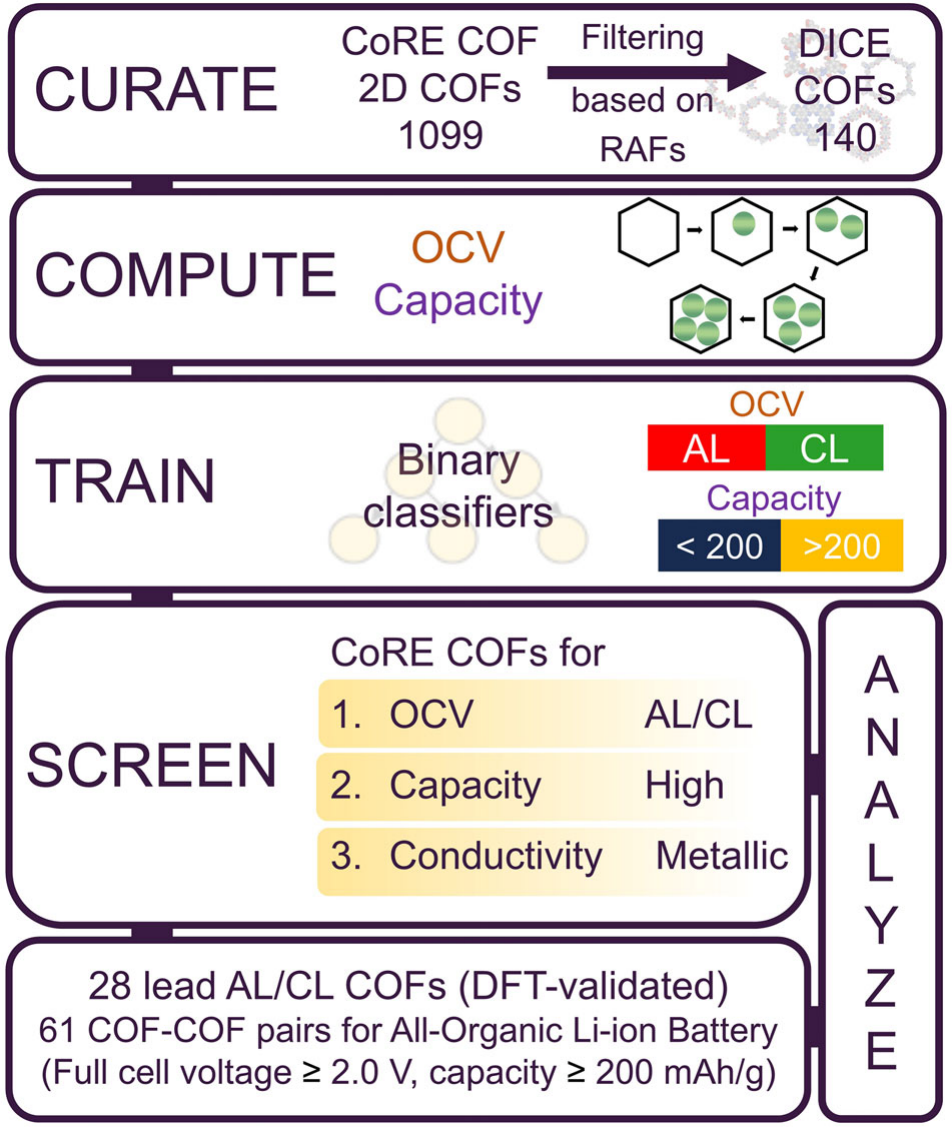
Overview of the workflow for classifying and identifying potentially high‐performing electroactive COFs. AL and CL correspond to anode‐like and cathode‐like behavior of COFs. Electronic conductivity is estimated from the first stage of Li^+^ insertion.

In this research direction, the current literature reports larger number of COF anodes as compared to cathodes, while the average reported capacity values span over a wide range (40–1200 mAh g^−1^) [32, 37, 38, 39, 40, 41, 42, 43, 44, 45, 46, 47, 48, 49, 50, 51, 52, 53, 54, 55]. These studies typically focus on synthesizing and testing one or two targeted COF families by varying functional groups, pore size, crystallinity/stacking, or layer thickness. While such investigations establish proof‐of‐concept performance, they collectively fall short of establishing robust design principles that link COF structure and chemistry to their potential electrode behavior (as determined by the voltage) and accessible capacities. An additional important characteristic is the electron/hole conductivity under realistic operating conditions. Assessing the suitability of 2D COFs as LIB electrode materials requires systematic evaluation across the thousands of synthesized COFs reported to date [56, 57], making experimental trial‐and‐error approaches alone clearly impractical. Computational screening offers a promising alternative, but it requires reliable estimation of the above‐mentioned key properties such as voltage, capacity, and electronic conductivity, for which brute‐force computations across thousands of candidate systems spanning multiple 2D COF families are nontrivial, highly challenging and extremely resource‐intensive. In this context, computational screening accelerated by machine learning (ML) approaches offers a powerful route to systematically explore the electrochemical properties and underlying structure–property relationships. ML has already been successfully employed in COF domains to analyze mechanical stability, electronic structure, gas adsorption, and storage and separation performance [58, 59, 60, 61, 62] and in the field of MIBs to predict voltage [63], ion migration barriers [64], electrode performance [65], and electrolyte compatibility [66], novel organic Li‐ion battery cathodes [67], and Li‐ion transport mechanism in metallosalen COFs [68]. However, ML models for predicting electrochemical properties of 2D COFs from their structural and chemical features remain notably scarce [69].

In this work, we establish structure‐electrode‐function relationships across thousands of synthesized 2D COFs to assess their potential toward a viable replacement of conventional electrodes in LIBs. To this end, we develop a data‐driven framework by integrating density‐functional theory (DFT) computations and ML models to screen COFs based on three key criteria, namely their open‐circuit voltage (OCV), theoretical capacity, and electronic conductivity. Our strategy involves curating a chemically diverse subset of synthesized 2D COFs from the CoRE COF (a comprehensive collection of 1099 **Co**mputation‐**R**eady **E**xperimental COFs) database [70], followed by labeling this curated set with DFT‐derived OCVs and accessible theoretical capacities. Based on these labeled datasets, we train ML binary classifier models that distinguish, (1) potential anodic and cathodic behavior, and (2) COFs with substantially high capacities. These ML models enable us to screen CoRE COF database to pinpoint 28 lead candidates; 14 anodes (0.26–0.62 V, 200–577 mAh g^−1^) and 14 cathodes (2.00–2.93 V, 275–440 mAh g^−1^), identified through sequential voltage, capacity and metallic‐character‐based filtering, and finally, validated by explicit DFT calculations. Our analysis establishes concrete atomic‐level design rules that rationalize and predict anode vs. cathode‐like behavior of 2D COFs in terms of local coordination environment, heteroatom proximity, and unit‐cell features. Based on a direct comparison with conventional inorganic electrodes, we conclude that 2D CoRE COFs are not straightforward drop‐in replacements for conventional LIB electrodes. However, we highlight that they are highly attractive for sustainable AOLB technologies, where optimally designed COF–COF pairings, guided by our design rules, can surpass the current state‐of‐the‐art.

## 2 Results and Discussion

### 2.1 Data Curation and Labeling With DFT‐derived OCV and Capacity

Our starting point is the CoRE COF database, which contains a comprehensive collection of 1099 crystal structures of experimentally synthesized COFs. We have first removed (i) duplicate entries, (ii) COFs containing metal centers, and (iii) disordered structures to obtain an updated list of 996 COF entries and subsequently, curated a diverse set of 140 COFs. By thoroughly examining prior experimental and theoretical studies [19, 20, 21, 22, 23, 24, 25, 26, 27, 28, 29, 30, 31, 32, 33, 34, 35, 36, 37, 38], we have selected these COFs to ensure diversity in building blocks, connectivity, and, most importantly, the type and accessibility of RAF sites that govern metal‐ion binding and insertion thermodynamics, which directly determine the OCV, and achievable capacity. The curated collection of 140 COFs (hereby denoted as **D**iverse **I**nventory of CoRE **C**OFs for **E**lectrodes or DICE) spans the major RAF chemistries commonly explored in electroactive COFs, including carbonyls (quinones, ketones, imides), imines family (azines, hydrazones, phenazines), nitro groups, macrocycles (porphyrins, phthalocyanines), and other electron‐deficient (triazines, heptazines) motifs.

For each of these selected 140 2D COFs, we have computed the OCV and gravimetric capacities using a consistent computational workflow as depicted in Figure 2a. To briefly illustrate this procedure, we take COF‐461 (Tb‐DANT‐COF) as the representative example [71]. For each Li^+^ adsorption site (colored bubbles in Figure 2b), we first compute the Li^+^ binding energy using DFT at the PBE‐D3(BJ) level of theory and rank‐order the sites. Next, we begin lithiation from the most favorable site and then proceed sequentially to less favorable sites until either all accessible sites are occupied or further lithiation becomes thermodynamically unfavorable. We analyze the resulting theoretical voltage profile (Figure 2b) and estimate (i) OCV from the first insertion step, which we use to classify COFs as anode‐like or cathode‐like, and (ii) capacity from the final stage with the highest Li^+^ content. Applying this workflow on all the DICE COFs leads to two datasets of 2D COFs labelled with the corresponding OCV and capacity values, which we use for training ML models for downstream prediction of these electrochemical properties. We denote the datasets as DICE‐V (for OCV classification) and DICE‐C (for capacity classification), each containing 137 2D COFs. Note that 134 out of 137 entries are common between the two datasets while the remaining three entries are different depending on the computational feasibility of the DFT derived OCV and capacity values. The dataset is publicly available at https://doi.org/10.5281/zenodo.21000393.

**FIGURE 2:**
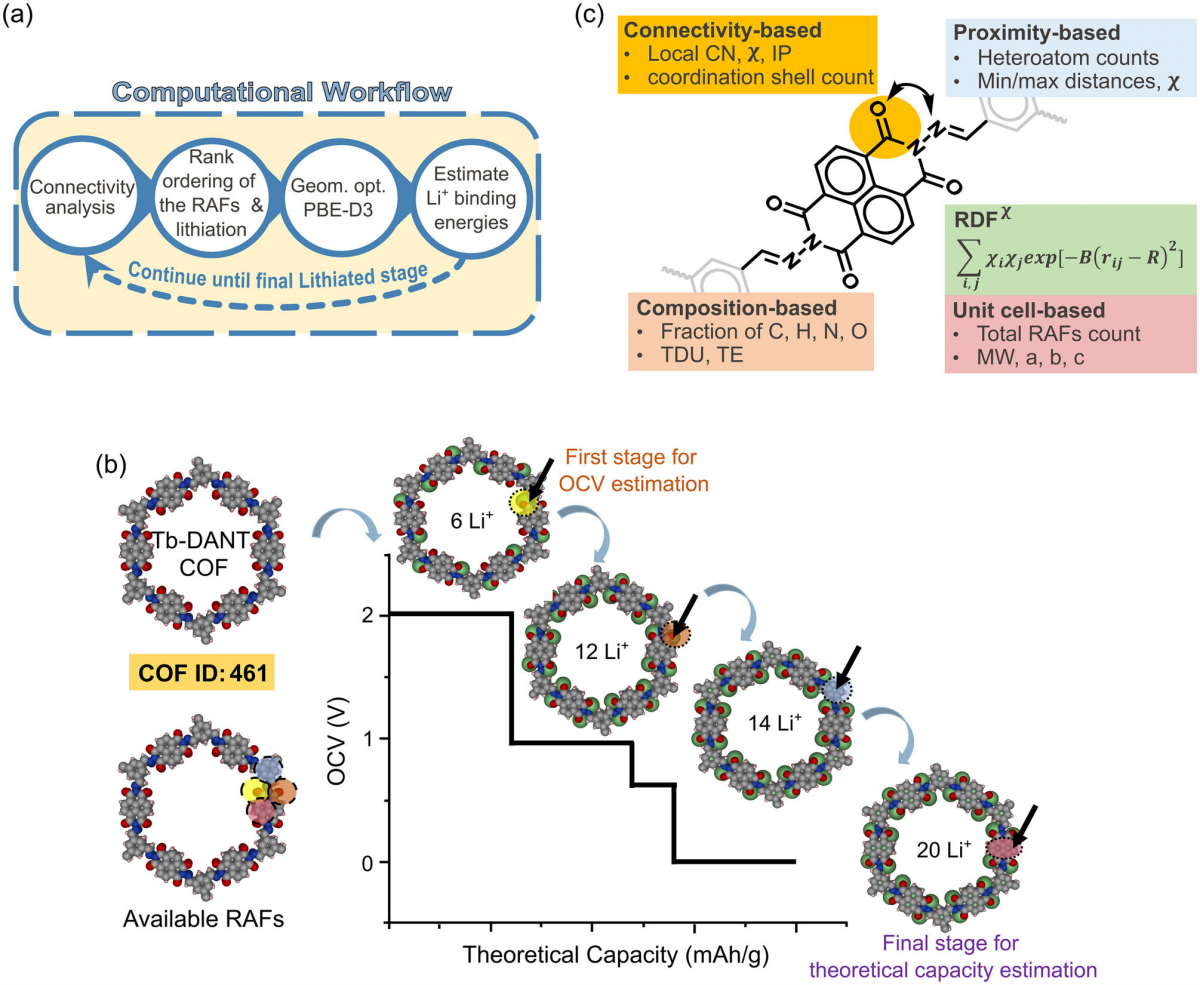
(a) Schematic overview of the computational workflow for the theoretical estimation of the OCV and the capacity for the selected 140 2D COFs. The final lithiated stage implies either that all accessible sites are occupied or that the next insertion becomes thermodynamically unfavorable. (b) Illustration of the computational workflow using a representative COF (CoRE COF ID 461). Grey, pink, red, blue, yellow, and green spheres represent C, H, O, N, S, and Li atoms, respectively. (c) Schematic overview of the COF featurization strategy for the OCV and capacity class prediction.

### 2.2 COF Featurization

After setting the labels, we have utilized the unrelaxed Crystallographic Information Files (CIFs) for featurizing the 2D COFs to be used as inputs to the ML models for electrode behavior predictions. We extract thirty‐one composition‐based and local features to encode the structural and chemical characteristics of the unit cell of each COFs for the OCV class prediction task (see Figure 2c and Table S2). The composition‐based features include six elemental composition ratios (fractional C, H, N, O, N+O, and halogen contents) along with two derived properties; the total degree of unsaturation (TDU), and the total count of electronegative atoms (TE). The rest of the twenty‐three local features capture the specific chemical environments associated with the RAFs and are subdivided into three groups; (i) eight connectivity‐based features defined as mean aggregates of key atomic and coordination properties around the sp^2^ O and sp^2^ N sites (or aromatic rings in their absence), including local coordination number, electronegativity, ionization energy, and coordination shell statistics, (ii) five proximal features quantifying the nature and proximity of nearby heteroatoms (N, O and S), describing their count, electronegativity, and spatial distance, and (iii) ten features derived from the electronegativity‐weighted radial distribution functions encoding the spatial correlations of these proximal heteroatoms, capturing structural organization relevant to the redox behavior. Five additional features, including the three unit cell dimensions, number of total sp^2^ O and N sites, and the molecular weight of the unit cell are additionally used for training models for accessible capacity prediction. A complete list of features with additional details is available in the Table S2.

### 2.3 Diversity Analysis

Before training the ML models, we explore the diversity of the 137 COFs in the DICE‐V dataset based on their features and theoretically derived OCV and capacities. To this end, we have created a 2D representation of the chemical diversity of the CoRE COF database using a t‐distributed stochastic neighbor embedding (t‐SNE) map [72]. The clustering of different CoRE COFs (grey points in Figure 3a) is based on their chemical and geometrical environments. The overlay of DICE‐V structures indicates that this collection of COFs samples the parent dataset without overly representing a specific COF family and therefore, does not introduce significant chemical domain bias. This is further evidenced by the broad distributions of computed OCVs and theoretical capacities in the DICE‐V and DICE‐C datasets, respectively. As shown in Figure 3b,c, the values span from −0.27 to 2.71 V and from 34.7 to 700.0 mAh g^−1^ (mean: 1.0 V and 292.7 mAh g^−1^) for OCV (capacity), respectively, reflecting the wide chemical diversity of the datasets and their ability to capture a broad spectrum of electrochemical behavior. Furthermore, to assess the reliability of the predictions for the screened CoRE COFs, we have evaluated the applicability domain with respect to the DICE COF training set (Section S1 and Figure S2). This analysis shows that 94.7% of the remaining CoRE COFs lie within the descriptor space represented by the training data, supporting the use of the trained models for screening the CoRE COFs.

**FIGURE 3:**
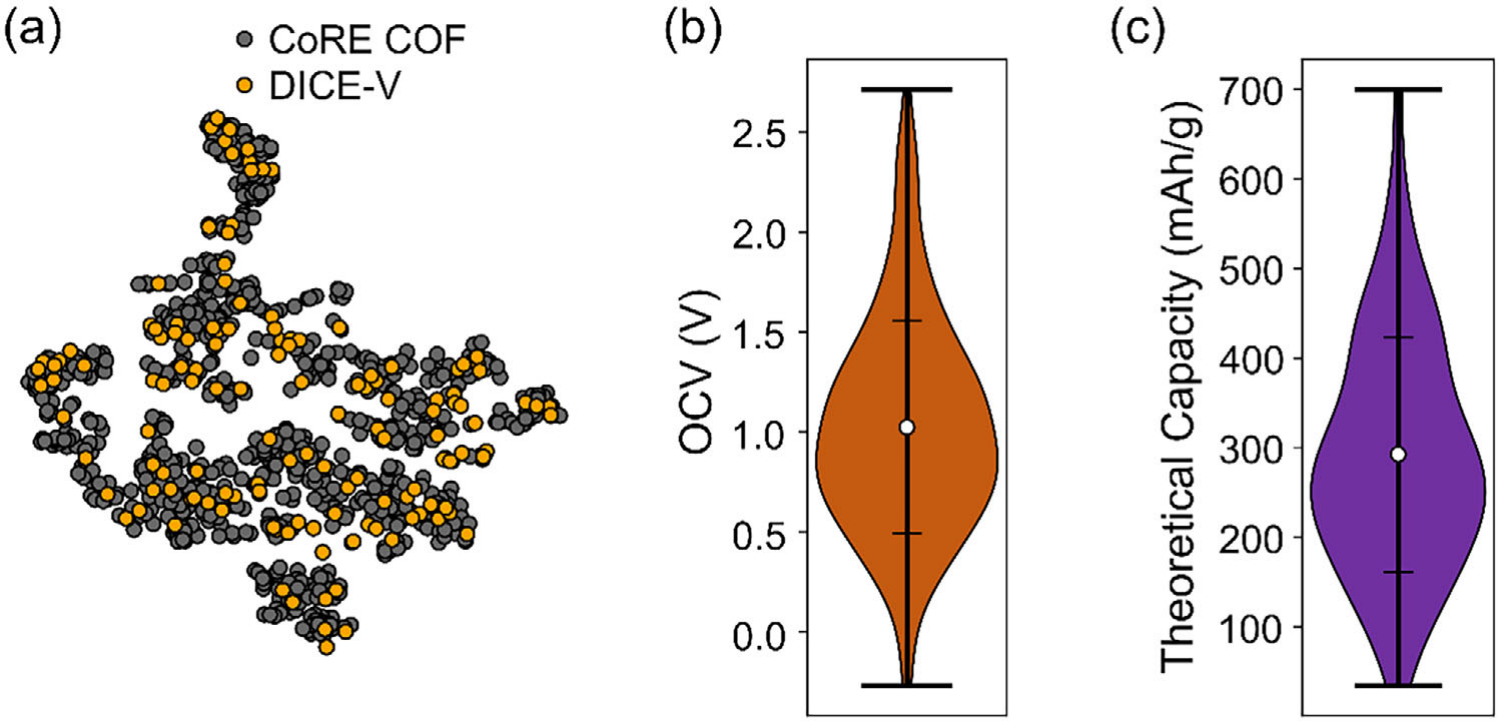
(a) 2D t‐distributed stochastic neighbor embedding (t‐SNE) map of the CoRE COF database (grey points). The DICE‐V dataset, used to train the ML models for OCV class prediction, is overlayed (yellow points). The embedding was generated using 31 compositional and local descriptors derived from the experimental crystal structures of the CoRE COFs. The distribution of the computed (b) OCV and (c) theoretical capacity values in the DICE‐V and DICE‐C datasets, respectively.

### 2.4 Training of ML Models for OCV and Capacity Class Prediction

#### 2.4.1 OCV Classification

We next train ML models to classify the potential electrode behavior of each COF in the DICE‐V dataset as anode‐like, when OCV < 1.6 V and cathode‐like, when OCV ≥ 1.6 V. The relative proportions of the anode‐like and cathode‐like COFs in the dataset are 88% and 12%, respectively, which makes this classification task non‐trivial. To handle the imbalanced distribution and limited size of the database, we have trained each model using stratified folds for train‐test dataset splitting to ensure the same ratio of anode‐like vs. cathode‐like labels between the two sets. The performances of all trained classifiers are evaluated using the area under the receiver operating characteristic curve (ROC‐AUC) and other performance metrics (please see Figure S3 for further details). A random classifier typically produces a ROC–AUC score of 0.5 (grey dashed line in Figure 4a), representing no discriminative power, whereas values closer to 1.0 reflect progressively improved model performance.

**FIGURE 4:**
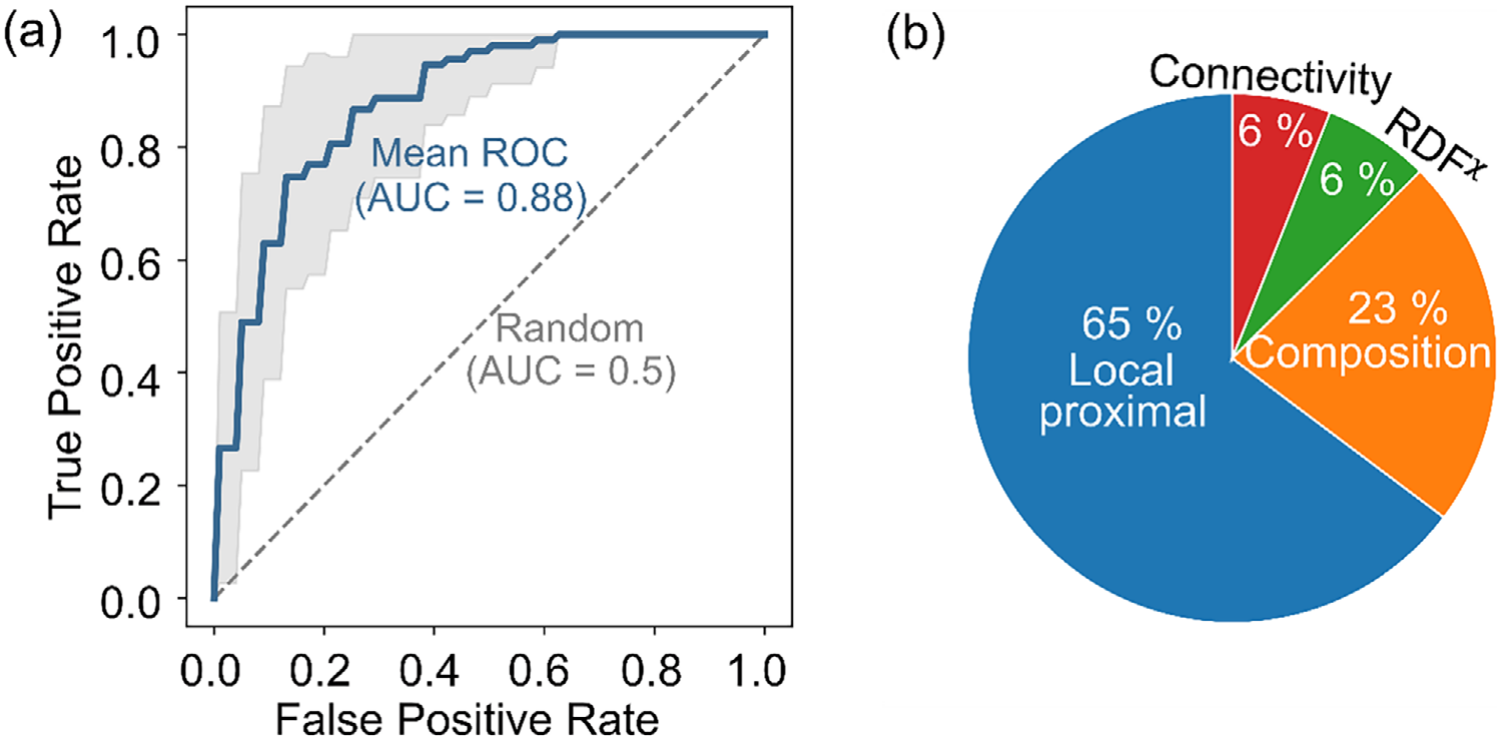
(a) Mean ROC curve describing the performance of the consensus‐based ensemble XGBoost classification model at various threshold values. The grey dashed diagonal line corresponds to the ROC curve expected from a random binary classifier. (b) Average feature importance values (converted to percentages) for local and composition‐based descriptors.

First, we evaluate several classification algorithms, including XGBoost, Random Forest, Support Vector Machine, Logistic Regression, and k‐Nearest Neighbors, for the electrode classification, and find that XGBoost achieved the highest performance based on the ROC–AUC and F1‐score (see Table S3, for a detailed comparison of the various performance metrics of different models). Subsequently, we construct a consensus‐based hard‐voting ensemble of five XGBoost classifiers from the top‐performing hyperparameter configurations to predict the final OCV class. Further details about the training of the classifiers are provided in Section S2. To assess the robustness of the predictive performance, we employ 5 × 5 repeated cross‐validation (CV), following the recommendation of Ash et al., where five‐fold stratified CV is repeated five times using different data partitions [73]. For each fold, recursive feature elimination (RFE) is carried out on the corresponding training partition using an inner five‐fold stratified CV. The XGBoost ensemble is then trained using the selected features and evaluated on the held‐out test partition. Averaged across 25 test partitions, the resulting OCV classifier achieves a ROC‐AUC of 0.88, and balanced accuracy of 0.77. To account for the class imbalance, we additionally evaluated the precision‐recall AUC (PR‐AUC), which is more sensitive to performance on the minority class (cathode‐like COFs) than ROC‐AUC, using the pooled out‐of‐fold predictions within each of the five CV repetitions. The model achieves a mean PR‐AUC of 0.51, substantially exceeding the random baseline defined by the minority class prevalence 0.124.

The subsequent feature importance analysis shown in Figure 4b and Figure S4 reveals that local features from the redox sites dominate OCV classification, contributing approximately 77% of the total importance. The dominant contribution of the proximal features (65%) highlights the critical role of the proximity of nitrogen and oxygen heteroatoms within the local environment of the RAFs in determining the electrode behavior. In contrast, composition‐based features, such as the ones that reflect the nitrogen and oxygen content of the COF frameworks, account for 23% of the overall importance. To assess the robustness of this interpretation, we have further evaluated feature selection frequencies across the 25 outer CV folds and performed grouped permutation importance analysis on the held‐out data (Section S3). We find that across different data splits the dominant proximal and compositional features such as mean_en, max_en, mean_num, and n_NO are selected consistently (100%) followed by other important features such as max_d (92%) (Table S4). Furthermore, consistent with the feature importance analysis (Figure S4), both proximal and composition‐based features showed the most robust contributions to predictive performance supporting the predominant role of local chemical environments of the RAFs in the model predictions (see Figure S5).

Furthermore, we have performed additional analysis to understand which features are most predictive of determining electrode behavior (Figure S6). We find that some features make the model more likely to classify a COF as cathode when the feature value is high, such as the *mean_en* and *max_en* features for the OCV classification. These features encode the electronegativity of the atoms in the proximal environments of RAFs, and a higher value indicates the presence of electronegative atoms such as N, O, and S, offering multiple contacts to Li, like in acylhydrazone linkages, keto‐amine, imide cores, etc. The distributions of these features are significantly shifted to higher values for cathode‐like COFs in comparison to anode‐like COFs. Similarly, the mean_num distributions for cathode‐like COFs are shifted toward higher values compared to the anode‐like COFs. On the contrary, the n_NO, which encodes the collective nitrogen and oxygen contents of the COFs, does not show markedly different distributions, indicating this has a lower impact on deciding the electrochemical behavior. We emphasize that the observed feature‐distribution trends indicate correlations rather than causal relationships and may partly reflect the specific composition of the DICE COF dataset. Nevertheless, the substantial overlap between the chemical spaces represented by the DICE and CoRE COF datasets suggests that these trends may be transferable to the latter, although their generality beyond the training domain should be interpreted with caution.

Together, these findings underscore that both local structural factors and overall chemical composition collectively influence the redox behavior, with the former being the primary determinant.

#### 2.4.2 Capacity Classification

Next, we develop an additional binary classifier to predict whether a COF candidate can achieve a capacity exceeding 200 mAh g^−1^. This cutoff, set as the approximate average of the reported theoretical capacities of commercial cathodes LiCoO_2_ (274 mAh g^−1^) [74] and LiFePO_4_ (174 mAh g^−1^) [75], is chosen to preferentially retain all potentially promising cathode‐like materials in the screening. This capacity prediction model is trained using the DICE‐C dataset, following the same ML architecture as the OCV classification workflow (Section S2). The final capacity class prediction is thus based on a consensus‐based hard voting XGBoost ensemble with a RFE‐reduced feature set (Figure S7). The model yields ROC‐AUC and balanced accuracy values of 0.75 and 0.67 (from a 5 × 5 repeated stratified CV), respectively, indicating moderate discriminative performance and therefore, serves only as a preliminary filter to prioritize COFs expected to exhibit high capacity. In contrast to the OCV classification model, we find that the unit cell features, especially the c‐axis parameter, dominate the prediction (Figure S8). This indicates that capacity is governed by the total number of Li^+^ ions that can be hosted within RAFs, interlayer galleries, and pores. As discussed in the DFT lithiation workflow in Section 2.1, Li^+^ intercalation into the interlayer spaces is a critical step that is especially sensitive to the c‐axis parameter. Compositional descriptors, particularly those describing nitrogen and oxygen content, also play a notable role, whereas local proximal (redox‐site) features exhibit comparatively lower relevance in determining capacity. The robustness of these predictions is further supported by determining the selection frequencies and grouped permutation importance of the features and these are reported in Table S5 and Figures S9 and S10.

### 2.5 ML Prediction of Electrode Suitability Across Entire CoRE COF Database

#### 2.5.1 Structure‐OCV Relationships in COFs

Next, we have applied the trained OCV classifier across the remaining candidates of the CoRE COF database. Figure 5a shows the 2D t‐SNE projection of the 2D COF structures colored by their predicted electrode behavior, cathode‐like in red and anode‐like in blue. 85% of 2D COFs are classified as anode‐like, while only 15% as cathode‐like. We analyze the clusters that are predominantly predicted to exhibit cathode‐like (CL1‐CL7) and anode‐like behavior (AL1‐AL4) to reveal distinct geometric and chemical signatures underlying this classification (Figure 5b,c). Examination of their local environments indicates that cathode‐like behavior arises in frameworks featuring dense, multidentate RAF pockets accessible at the pore walls as potential Li^+^ binding sites (Figure 5b). Typically, cathode‐like COFs should provide three or more Li^+^ coordination contacts per local redox environment, involving nitrogen or oxygen heteroatoms from the RAF sites. As clearly seen from Figure 5d–f, two of the donor sites are typically arranged in‐plane at a suitable distance of 2.5–2.8 Å, while the third (or additional) donor arises either from interlayer periodicity (3.2–6.7 Å) or an appropriately oriented side group (at a distance of ∼2.5–3.0 Å). Therefore, the desirable chemical motifs to predict a COF with cathode‐like behavior are proximate N/O ensembles as exemplified by acylhydrazone linkages (CL1), keto–amine environments (CL2‐CL3), often augmented with electron‐withdrawing substituents such as –SO_3_H, –NO_2_, and –COOH (CL3), and imide cores (CL4 and CL7) that enhance Li‐host interaction strength and increase the redox potential. Likewise, nitrogen‐rich frameworks produced by aza‐extended aromatics (e.g., hexaazatriphenylene in CL6) promote multi‐Li coordination through 4 Li–N contacts (Figure 5e), while incorporation of π‐macrocyclic motifs such as porphyrins from CL5, further elevates voltage by offering a preorganized N_4_ coordination pocket within a conjugated scaffold (Figure 5g).

**FIGURE 5:**
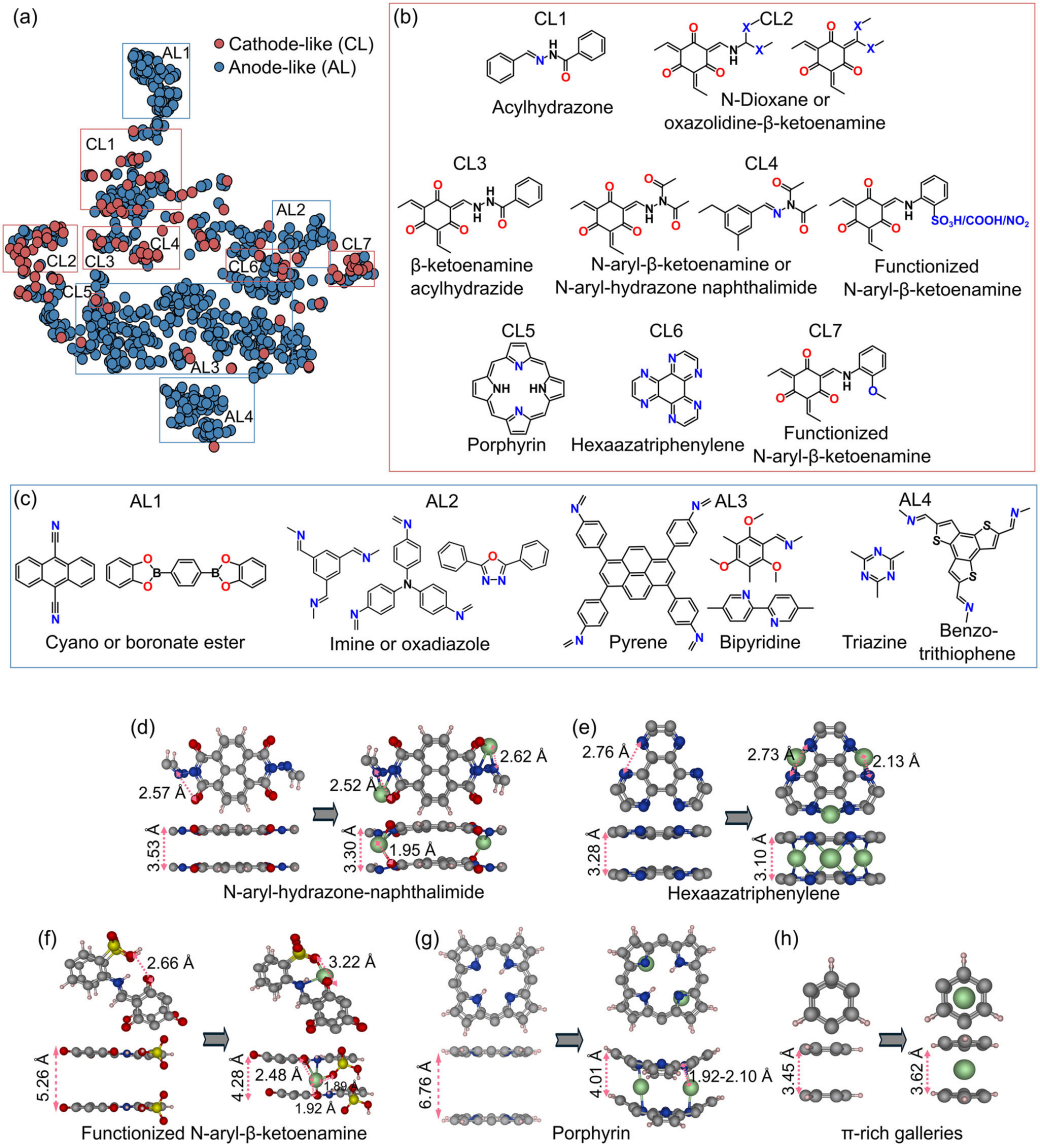
(a) 2D t‐distributed stochastic neighbor embedding (t‐SNE) map of the CoRE COF database based on predictions from the OCV classifier trained on the DICE‐V dataset, where the red and blue balls represent cathode‐like and anode‐like COFs, respectively. The DICE‐V entries are removed from the plot for visual clarity. Examples of chemical motifs exhibiting (b) cathode‐like (CL) and (c) anode‐like (AL) behavior. The embedding was generated using 31 compositional and local descriptors derived from the experimental crystal structures of the CoRE COFs. (d‐h) Optimized Li^+^‐bound geometries of some selected structural motifs, representative of various clusters in the t‐SNE map. The redox active groups involved in Li^+^ binding are shown by red and blue colors. Fragments of selected 2D COF candidates illustrating changes in interlayer spacing, in‐plane and interplane binding‐site separations, and the corresponding Li–N/O distances. Grey, pink, red, blue, yellow, and green spheres represent C, H, O, N, S, and Li atoms, respectively.

In contrast, our analysis of predicted anode‐like candidates (Figure 5c) reveals the opposite trend, featuring weaker‐to‐moderate Li^+^ binding that enables facile (de)intercalation at low potential. As demonstrated by AL1‐AL4, two molecular architectures emerge as characteristics of this anodic behavior. First, frameworks with spatially separated redox sites prevent multidentate chelation, maintaining shallow Li^+^ binding. In this regime, imine, triazine, pyridine, and five‐membered heteroaromatics (e.g., pyrrole, oxadiazole, etc.) serve as suitable primary functionalities when kept spatially separated, as observed across AL1–AL4. Second, intercalation within interlayer π‐rich galleries enables Li–π interactions with extended pyrene (AL3), phenyl, heteroaromatic surfaces (boronate ester (AL1) and benzothiophene (AL4), facilitating reversible uptake without strong chemisorption. This Li^+^ insertion mode typically sets the interlayer spacing of the 2D COFs to ∼3.7 Å as illustrated in Figure 5h.

These derived structure‐electrode functions are consistent with experimental observation as shown in Section S4 and Table S6. The imide COFs (p‐DANT‐COF [71], TPDA–PMDA [32]), hexaazatriphenylene COFs (HATN‐CMP [76], HATNPF1‐F2 [77]), functionalized β‐ketoenamine COFs (COF‐TRO) [78] and amide COFs (BTH‐NT COF) [79] have been reported as cathodes with voltage range of 1.5–4.0 V. Similarly, imine COFs (PA‐COF/TB‐COF [80], COFs@IM [81]), pyrene‐based COFs (PyTTF‐COF) [82], and triazole‐based COFs (IISERP‐CON1) [46] are all predicted as anode with low‐voltage window (0.02–1.45 V).

#### 2.5.2 Structure‐Capacity Relationships in COFs

The predicted capacities across the remaining CoRE COFs (Figure S11) shows that about 75% of the candidates are expected to have capacities exceeding 200 mAh g^−1^. When colored according to their predicted accessible capacity, the 2D‐tSNE map from Figure 5a (see Figure S11) indicates that most predicted low‐capacity 2D COFs belongs to cluster AL1 (e.g., COF‐48, 305, 306, 436, 182, 470, 101, 102, 350, etc.) containing redox‐inactive boronate ester linkages. This is attributed to Li^+^ intercalation being largely confined to the π‐acidic galleries formed between two electron‐withdrawing B–O units (Figure S12), resulting in intrinsically low capacities. Even more critically, boroxine‐based 2D COFs in cluster AL1 (e.g. COF 87 in Figure S13a) are essentially electrochemically inert and we recommend that these COFs should be avoided as electrode candidates. Additional trends emerge from clusters AL2 and AL3: in cluster AL2, low‐capacity 2D COFs typically feature electron‐donating groups such as ethers or flexible oligomer chains (COF‐938, 945, etc., Figure S13b), while in cluster AL3, COFs often feature triarylamine units, whose propeller‐like phenyl rings (Figure S13c) do not participate in Li^+^ intercalation.

### 2.6 Electron Conductivity‐based Screening on ML Predicted Electrodes

Based on the confidence of the ML predictions of OCV and capacity classes across the CoRE COF database, we retain an initial pool of 164 cathode‐like and anode‐like candidates, (Tables S7 and S8) for further analysis, which also includes the DICE COFs. We already have the DFT‐computed OCV for DICE, so for remaining COFs, we explicitly compute their OCV values using DFT and apply a stricter OCV threshold, OCV ≤ 0.62 V for anodes and OCV ≥ 2.0 V for cathodes, to further narrow down the pool to 17 anode and 14 cathode candidates to reach as close as the practical voltage windows of conventional electrode materials (e.g. LiCoO_2_, NMC, graphite and silicon (Si), etc., see Figure 6a) [74, 75, 83, 84, 85, 86, 87, 88]. Since electronic conductivity is a critical practical requirement for electrode performance, we next screen these 31 COFs by computing their density of states at HSE06 level (see computational methods and Figure S14). Specifically, we assess whether a COF undergoes metallization (band gap ≈ 0) at the first lithiation stage because this indicates enhanced electronic conductivity, beneficial for charge transport and rate performance [89, 90], and 28 COFs listed in Table S9, intrinsically satisfy this criterion. We note that Tp‐DANT‐COF (COF‐538) [71], Tb‐DANT‐COF (COF‐461, which is included in the DICE) [71], and DABQ‐TFP‐COF (COF‐199) [22], identified in this list as a cathode candidates with a predicted OCV of 2.30, 2.00, and 2.65 V has been experimentally demonstrated as a Li‐ion cathode with respective voltage plateau of ∼2.50, 2.50, and 2.80 V, and stable cycling and good rate performance. While the remaining three COFs are semiconducting after lithiation, we envisage that they could still be viable electrodes through extrinsic strategies of improving conductivity (Table S9) [15, 16, 17, 33, 91]. Overall, we observe that even though pristine COFs are inherently semiconducting, partial lithiation (first‐stage) can trigger semiconductor‐to‐metal transitions and substantially improve electron conductivity under battery operating conditions, consistent with previous studies [18, 32, 71, 87, 92]. Please note that these are pure theoretical predictions and a further experimental realization is necessary to confirm them. The practical behavior of these COF electrodes will also depend on factors other than OCV, capacity and electronic conductivity, such as rate capability (Figure S15) synthesis feasibility, SEI formation, cycle stability, electrolyte compatibility, etc.

**FIGURE 6:**
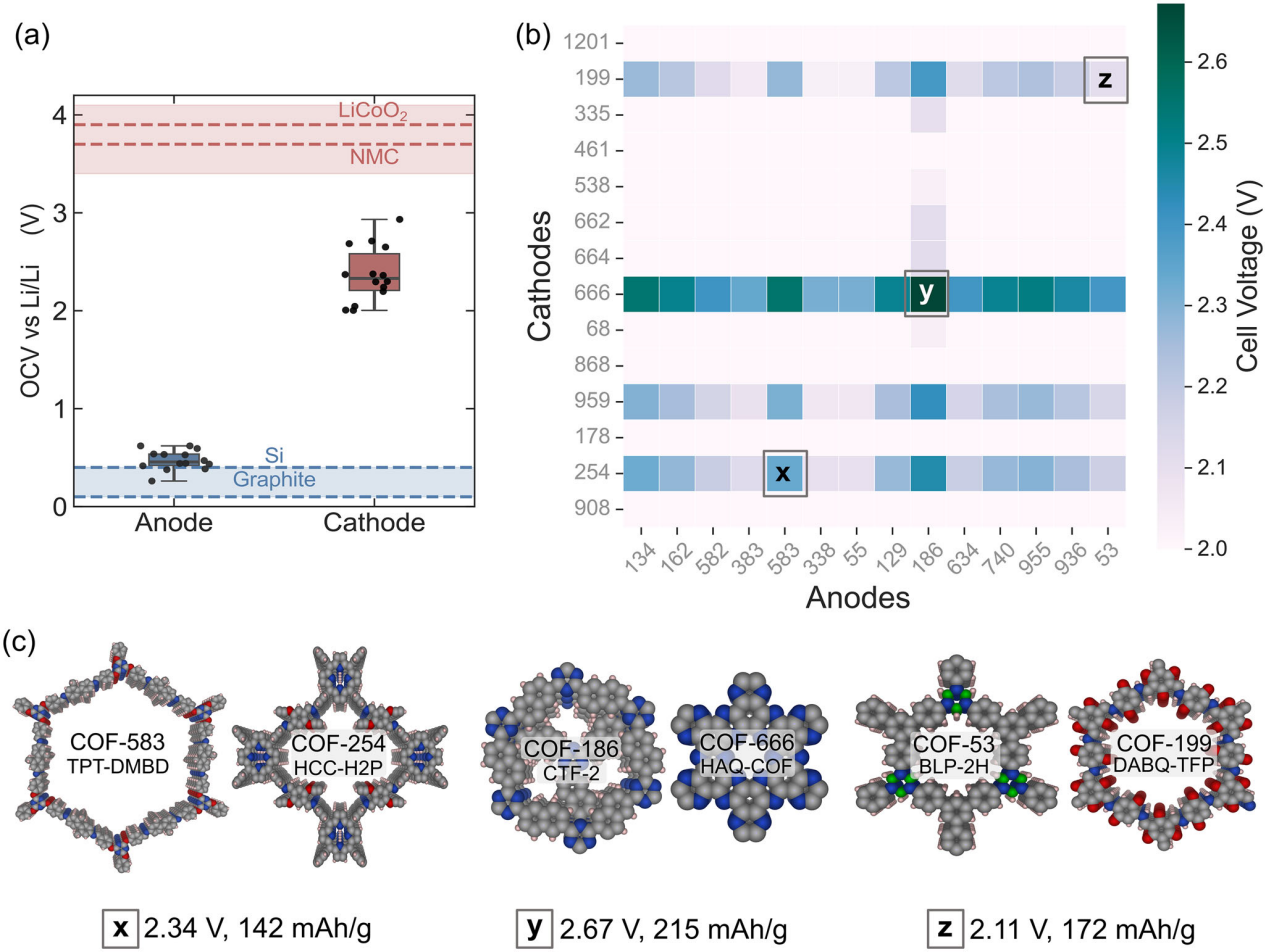
(a) Box plot for the computed OCV values of the 28 lead anode and cathode COF candidates derived from DFT calculations in comparison to those of conventional electrode materials. Shaded light‐blue and light‐red regions represent the voltage windows of graphite/Si anodes and NMC, LiCoO_2_, and LiFePO_4_ cathodes, respectively. (b) Heatmap showing the top‐performing anode‐cathode COF pairings for all‐organic lithium batteries (AOLBs) with full‐cell voltages surpassing 2.0 V. x, y and z correspond to three represented COF pairs. (c) Structural depiction for the selected anode|cathode full cell examples along with their DFT calculated full cell voltage and capacities.

### 2.7 Comparison with Conventional Electrodes and Practical Consideration for All Organic Li‐Ion Batteries

Having identified the 28 most promising COF electrodes within the chemical space of CoRE COFs, we finally, compare these results with commercial electrodes. As depicted in Figure 6a, the predicted 2D COF anodes span an OCV range of 0.26–0.62 V vs. Li/Li^+^ with theoretical capacities of 200–577 mAh g^−1^ (Table S10). Relative to graphite, which operates at 0.1–0.2 V vs. Li/Li^+^ and is prone to lithium plating during fast charging or low‐temperature operation, the screened COF anodes offer a potential reduction in Li‐plating tendency. In fact, we find that some of these COFs theoretically exceed graphite's capacity of 372 mAh g^−1^ [85, 86]. Compared to Si anode, these 2D COFs underperform both in capacity (∼3579 mAh g^−1^) and voltage suitability (0.3–0.4 V) [83, 84]. These porous structures can avoid ∼300% volume expansion that is observed for Si lithiation, however it requires further experimental or computational validation. For the cathode candidates, the screened COFs exhibit lithiation voltages that fall 1–2 V below those of prevalent inorganic intercalation cathodes: LiCoO_2_ (∼3.9 V, ∼274 mAh g^−1^) [74], LiFePO_4_ (∼3.4 V, ∼174 mAh g^−1^) [75], LiMn_2_O_4_ (∼4.1 V, ∼148 mAh g^−1^) [87, 93], and NMC (∼3.7 V, ∼200 mAh g^−1^) [88]. This implies that, despite achieving comparable or superior theoretical capacities of 275–440 mAh g^−1^, the COF cathodes are limited in operating voltage. This is attributed to the inherently weaker redox activity of organic N/O‐based functional groups compared to typical transition‐metal redox couples (e.g., Co^3^^+^/^4^^+^, Fe^2^^+^/^3^^+^, Mn^3^^+^/^4^^+^), benefiting from the significantly higher electropositivity of metal centers.

This comparison establishes that CoRE COFs, at their current stage of development, are not practical replacements as electrodes, especially as cathodes in conventional LIBs. However, this perspective changes when these 2D COFs are considered as both anode and cathode within a rocking chair AOLB architecture [3, 94, 95, 96]. The atomic‐level electrode design rules established in this work facilitates the identification of suitable COF‐COF pairings for AOLBs as presented in Figure 6b and Figure S16. Out of the 196 possible anode|cathode pairs in our pool, we identify 61 most promising combinations to furnish a full cell voltage of ≥ 2.0 V and capacity in the range of 101–290 mAh g^−1^ in their corresponding AOLB (colored box in Figure 6b and Table S11). Figure 6b,c depict three such representative structurally diverse anode|cathode pairs, (COF‐583|COF‐254), (COF‐186|COF‐666), and (COF‐53|COF‐199), resulting in full cell voltage 2.34, 2.67, and 2.11 V, full cell capacities 142, 215, and 172 mAh g^−1^, and full cell energy density of 333, 575, and 364 Wh kg^−1^, respectively. Overall, these 61 predicted pairs promise comparable or higher voltage, capacity and energy density performances than previously reported AOLBs with Li_4_TP|PTCDI‐DAQ (0.2–3.2 V, 229 mAh g^−1^, 296 Wh kg^−1^) [97], Li_2_TP|ABB_4_OLi (> 1.0 V, 120 mAh g^−1^, 216 Wh kg^−1^) [98], and Li_2_TPh|LiTCNQ (1.8 V, 150 mAh g^−1^, 270 Wh kg^−1^) [96]. In fact, the reticular chemistry of COFs offers inherent advantages for AOLBs over these small organic molecules and polymers electrodes, which are critically limited by electrolyte solubility, poor active‐site utilization, capacity degradation upon cycling, and high battery costs.

Beyond AOLBs, the screened COF anodes can be directly applicable as structurally stable, safer alternatives to lithium metal and graphite in a broader range of lithium battery configuration, including Li‐sulfur (Li‐S), Li‐selenium (Li‐Se), and Li‐oxygen systems. For COF cathodes, however, targeted functional group engineering is required to meet the distinct cathodic reduction chemistries of each system, polysulfide/polyselenide chain‐reaction in Li‐S and Li‐Se, and reversible Li_2_O_2_ deposition without cathode passivation in Li‐oxygen, underscoring the need for system‐specific optimization beyond the design rules established here.

## 3 Summary

In this work, we have developed a data‐driven framework integrating DFT computations and ML models to map the structure‐electrode‐function relationships across 1099 synthesized 2D COFs from the CoRE COF database. From a curated, chemically diverse subset of 140 COFs (DICE), we have trained XGBoost classifiers for distinguishing anode‐like and cathode‐like behavior based on OCV and predicting the accessible capacity range directly from chemical and structural descriptors derived from their experimental crystal structures. We establish that the electrode behavior is regulated by localized redox‐active environments, whereas the capacity is an outcome of unit‐cell parameters and total redox‐site density of COFs. We show that anode‐like behavior is shown by 85% of CoRE COFs, which is attributed to the spatially separated redox sites and interlayer π‐rich galleries leading to weak‐to‐moderate Li–π binding. On the contrary, cathode‐like behavior is associated with dense, multidentate N/O pockets offering three or more Li^+^ coordination contacts, as found in imide cores, acylhydrazones, keto‐amine motifs, and π‐macrocycles. We predict that 75% of CoRE COFs are associated with capacities above 200 mAh g^−1^, while boronate‐ester, boroxine, ether, and triarylamine motifs are identified as intrinsically poor Li^+^ storage units and should be avoided as electrodes.

By performing sequential screening with respect to three key criteria: voltage, capacity and metallic character; we identified 14 2D COF anodes (0.26–0.62 V, 200–577 mAh g^−1^) and 14 2D COF cathodes (2.00–2.93 V, 275–440 mAh g^−1^) as lead candidates among the CoRE COFs. While these candidates do not offer transformative improvements over their inorganic counterparts, and require further experimental exploration, their co‐deployment in an AOLB configuration are predicted to unlock a competitive performance. Our predicted COFs as anode|cathode pairings, guided by design rules developed herein, deliver full‐cell voltages of ∼2.0–2.67 V with capacities exceeding 200 mAh/g on both electrodes, surpassing existing small‐molecule and polymer‐based organic battery counterparts.

The screened 2D COF anodes are directly transferable to Li‐S, Li‐Se, and Li‐O_2_ architectures, while 2D COF cathodes will require further functional tuning to account for their cathodic reduction chemistries. Overall, the present framework offers a practical blueprint for extending ML‐assisted screening to other metal‐ion chemistries (Na^+^, K^+^, Mg^2^^+^, Zn^2^^+^) and framework families, accelerating rational organic electrode discovery.

## 4 Methodology

### 4.1 Computational Details for Theoretical Estimation of OCV and Capacity

The OCVs and capacities of COFs are calculated using DFT with Vienna Ab initio Simulation Package (VASP) [99, 100]. with projector augmented wave (PAW) pseudopotentials and the GGA‐PBE exchange‐correlation functional [101, 102, 103]. A plane‐wave energy cutoff of 520 eV is used and dispersion corrections are applied using DFT‐D3(BJ) [104, 105]. The total energy convergence criterion is set to 10^−4^ eV. Structures were fully relaxed until forces were below 10^−2^ eV/Å, with Brillouin zone sampling performed using a Γ‐centered, COF‐dependent k‐point grid. All input files with these parameters are available in the https://doi.org/10.5281/zenodo.21000393. The electronic band structures of the COFs are computed in VASP using the PBE functional and with VASPKIT to generate the KPOINTS path and to post‐process the results [106].

Within the theoretical framework, lithium intercalation, conversion or displacement into an electrode can be represented by the general equation (1) [107, 108],

(1) $$Li_{x_{i}}Electrode+\left(x_{j}-x_{i}\right)Li_{s}\to Li_{x_{j}}Electrode$$

where *x*_i_ and *x*_j_ are the limits of the intercalation reaction.

Because periodic DFT calculations are carried out on charge‐neutral supercells, the Li^+^/e^−^ pair is inserted computationally as a single neutral Li atom. Bulk BCC Li metal is used as a reference state for the neutral Li atom at 0 K and represents the combined chemical potential µ(Li^+^) + µ(e^−^). This is the standard convention corresponding to a Li‐metal counter/reference electrode and is used in computational modelling of batteries [107, 108]. Although the DFT calculation inserts a formally neutral Li atom, the physically inserted species is Li^+^, and the accompanying electron is not left delocalized in vacuum, it is transferred into the host electronic structure.

The average equilibrium voltage (OCV), *V*(*x*), is related to the difference in the Gibbs free energy, Δ*G*, between the delithiated phase (charged state) and lithiated phase (discharged state) by,

(2) $$V\left(x\right)\approx -\frac{\Delta G}{\left(x_{j}-x_{i}\right)F}$$

where *F* is the Faraday constant. Δ*G* can be approximated by the internal (potential) energy change per intercalated Li^+^ ion, since the vibrational and configurational entropy contributions to the cell voltage at room temperature are expected to be small. The change in total energy *E*, of the system from DFT leads to a predicted cell voltage that is an average value for compositions between *x_i_* and *x_j_* [107, 108],

(3) $$V\left(x\right)=-\;\frac{E\left(Li_{x_{j}}Electrode\right)-E\left(Li_{x_{i}}Electrode\right)-\left(x_{j}-x_{i}\right)E\left(Li\right)}{\left(x_{j}-x_{i}\right)e}$$

where, $E(Li_{x_{j}}Electrode)$ and $E(Li_{x_{i}}Electrode)$ are the electrode phase (in our case, COF) with *x_i_* and *x_j_* number of Li^+^ ions and is the energy of metallic (body centered cubic) Li.

The average theoretical capacity is estimated using the given equation (4),

(4) $$C=\frac{xnF}{M_{f}}$$

where *x* and *n* are the electronic charge and number of Li ion in the formula unit of the COF, respectively. *F* is the Faraday constant, and *M_f_* is the molecular mass of the formula unit.

And the full‐cell specific capacity for the potential COF pairs is calculated using,

(5) $$\;C_{cell}=\frac{C_{A}\;\cdot \;C_{C}}{C_{A}+C_{C}}\;$$

where, C_A_ and C_C_ are capacities of anode and cathode, respectively.

The reliability of our computational protocol is validated by benchmarking the calculated OCV and theoretical capacity against experimentally reported voltages and/or capacities of COFs as presented in Section S5 and Table S12.

### 4.2 ML Models

We have trained all ML models using scikit‐learn 1.6.1 [72]. The final predictions of the electrode behavior are performed with a hard‐voting ensemble of five XGBoost classifiers, each derived from the top‐performing hyperparameter configurations. In this consensus‐based approach, the final class prediction is determined by majority voting among the individual classifiers. For the final prediction task, we have applied recursive feature elimination (RFE) with cross‐validation (CV) which iteratively removes less important features while using cross‐validation to select the optimal feature subset. This lowers model complexity, mitigates overfitting, and improves interpretability, which is an especially important consideration here, given the relatively limited dataset size for the classification task. The complete pipeline is evaluated using 5 × 5 repeated stratified CV, in which five‐fold CV was repeated five times with different data partitions, resulting in 25 outer train‐test evaluations. For each outer fold, RFECV is performed exclusively on the corresponding training partition using an inner five‐fold stratified CV. The number and identity of the selected features were therefore allowed to vary across the outer folds. For each outer fold, the ensemble is subsequently trained on the corresponding training partition using the selected features and evaluated on the held‐out test partition. Thus, the samples in the outer test partition are not involved in the feature selection procedure. We have measured the performance of the trained classifiers using ROC‐AUC, balanced accuracy, and F1‐score. For ROC‐AUC, probabilistic outputs from the five constituent XGBoost classifiers were averaged to obtain the consensus class probabilities. Balanced accuracy and weighted F1‐score are calculated from the hard‐voting class predictions. Given the imbalance between the two classes, we additionally evaluated the PR‐AUC for OCV classification. Since individual test folds contain only a few positive samples, we have estimated PR‐AUC from pooled out‐of‐fold predictions within each five‐fold CV repetition. This yielded one PR‐AUC value per repetition, from which the mean and standard deviation across the five repetitions were calculated.

After training, we have performed a feature importance analysis by averaging feature importances across the five constituent XGBoost models from the ensemble to identify the key features contributing to the classification tasks. To assess the predictive contribution of different features, we have performed a grouped permutation importance analysis in which features are divided into groups according to their definitions in section 2.2 i.e based on composition, connectivity, proximal, and radial distribution functions and unit‐cell based. Within each outer CV fold, RFECV‐selected features belonging to a given group are jointly permuted 30 times in the held‐out test set, while the remaining features were unchanged. Grouped importance is quantified as the decrease in balanced accuracy relative to the unpermuted test set. The permutation values are averaged within each fold and subsequently across the relevant outer CV folds. More details about the construction of these models along with the details of the performance metrics can be found in Sections S2 and S3.

To evaluate the sensitivity of the classification results to the chosen decision boundaries, the complete model‐training and CV procedure was repeated using OCV thresholds of 1.5, and 1.7 V and capacity thresholds of 150 and 250 mAh g^−1^. The resulting models are evaluated using the same performance metrics as the original models and the results indicate that the overall classification performance is reasonably robust to moderate variation in the decision boundaries. The details of these analyses are provided in Section S6 and Table S13. Additionally, we performed a comparison of the OCV and capacity classification models when the features are extracted from the unrelaxed or relaxed CIFs of the COFs. While the performance metrics slightly change, the main conclusions from the feature importance analysis were preserved (see Table S14).

To assess the applicability domain of the trained models, we have performed a nearest‐neighbor analysis using features which were standardized using the mean and standard deviation of the DICE‐V, and the same transformation is applied to the rest of the CoRE COF entries. First, within the DICE‐V, for each COF, the Euclidean distance to its nearest neighboring COF was calculated, excluding itself, and the 95th percentile of these distances is used as the AD threshold. Next, each of the remaining COFs from CoRE is then assigned its distance to the nearest DICE COF and considered within the applicability domain when this distance is below the defined threshold. Further details of this analysis are provided in Section S1.

As an additional validation of the OCV classifier, we randomly selected eight COFs from the CoRE COF (without the DICE entries) dataset without consideration of their ML‐predicted classes. Their OCV values were calculated explicitly using the same DFT protocol employed for the labelling of the DICE COFs. All eight structures were classified as anode‐like based on their computed OCV values, in agreement with the ML predictions (see Table S15). We note that the randomly selected subset contained no DFT‐validated cathode‐like structures. Given the predominance of the anode‐like COFs in the dataset (around 85%), the absence of cathode‐like structures in this randomly drawn subset is not unexpected. Consequently, this blind test provides additional validation of the anode‐like predictions but does not independently assess cathode‐like classes.

## Author Contributions

**Preeti Bhauriyal**: conceptualization, investigation, funding acquisition, writing – original draft, methodology, writing – review and editing, data curation, formal analysis, validation, visualization. **Shubhajit Das**: conceptualization, investigation, funding acquisition, writing – original draft, methodology, validation, writing – review and editing, data curation, formal analysis, visualization. **Thomas Heine**: writing – review and editing, methodology, conceptualization, supervision, resources, project administration, funding acquisition.

## Conflicts of Interest

The authors declare no conflicts of interest.

## Supporting information




**Supporting File**: advs78599‐sup‐0001‐SuppMat.pdf.

## Data Availability

The input and output files of DFT calculations are available on https://doi.org/10.5281/zenodo.21000393. The code used for training the classifiers for OCV and capacity predictions, DICE‐V and DICE‐C datasets are available on GitHub at https://github.com/dosshubha/COF‐ML along with the updated CORE COF database list after removing (i) duplicate entries (ii) COFs containing metal centers and (iii) disordered structures.
