# Supplementary material for: When are Two‐Dimensional Covalent Organic Frameworks Good Electrodes for Lithium Ion Batteries? A Data‐Driven Analysis
Source: Adv Sci (Weinh). 2026 Nov 1:e78599. Online ahead of print. doi: 10.1002/advs.78599 (PMC13723728; doi:10.1002/advs.78599)
Supplement: Supplementary file 1 [file ADVS-9999-e78599-s001.pdf]

Supporting Information

When Are Two-Dimensional Covalent-Organic Frameworks Good Electrodes for Lithium Ion Batteries? A Data-Driven Analysis

Preeti Bhauriyal,[a,*,⊥] Shubhajit Das,[a,⊥] and Thomas Heine [a,b,c,*]

[a] Faculty of Chemistry and Food Chemistry, Technische Universität Dresden, Bergstrasse 66c, 01062 Dresden, Germany
[b] Department of Chemistry & ibs of nanomedicine, Yonsei University, Seodaemun-gu, Seoul 03760 Republic of Korea
[c] Ewha Womans University, Department of Chemistry, 52, Ewhayeodae-gil, Seodaemun-gu, Seoul 03760, Republic of Korea

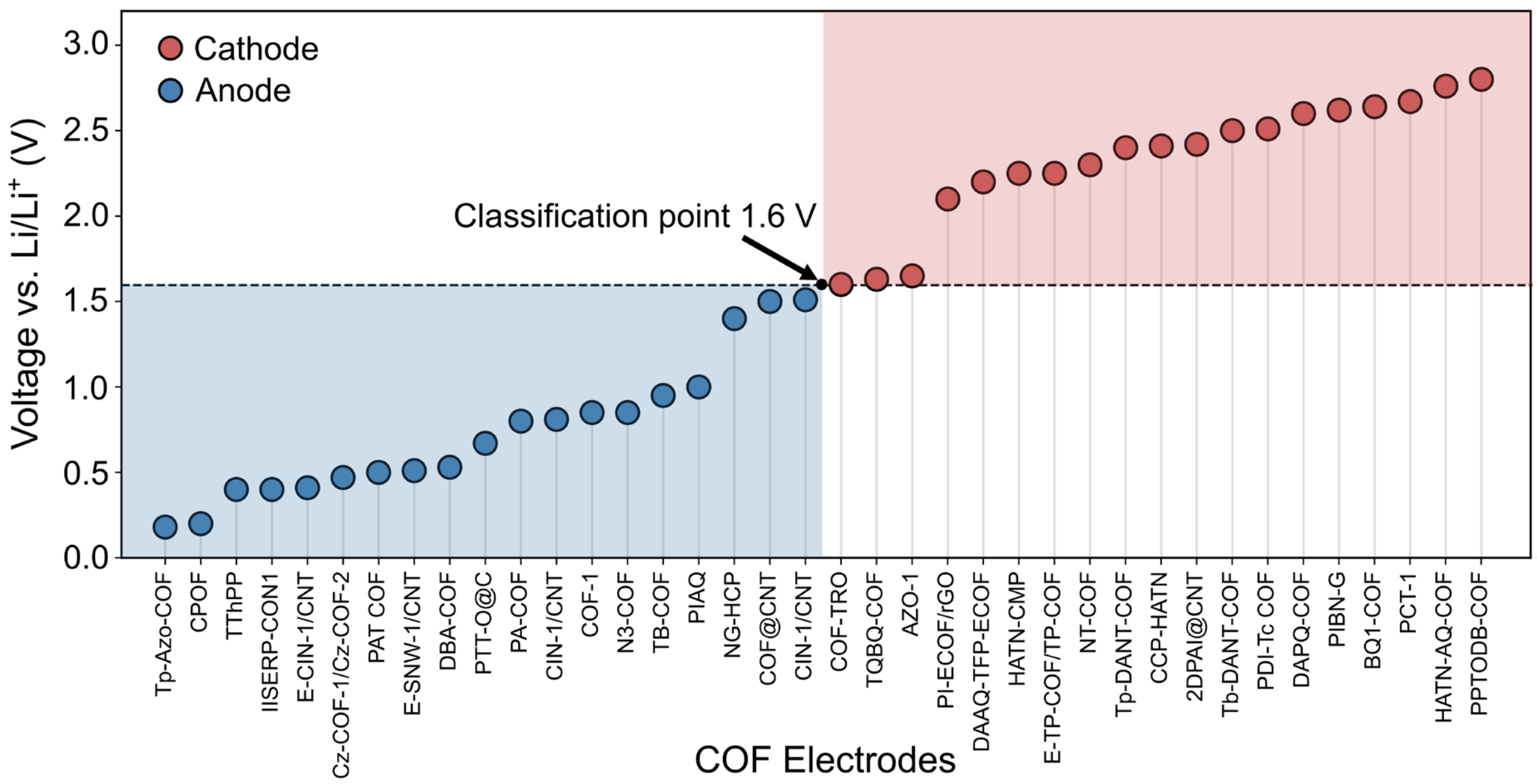

Figure S1: Experimentally reported voltage distribution of COFs as anodes and cathodes. Based on the observed trend, a vertical boundary is drawn at 1.6 V, differentiating the anodic or cathodic behavior. The COF electrodes are listed in Table S1, Supporting Information, along with the respective references.

Table S1: Voltage distribution of experimentally reported COF anodes and cathodes.

S. N.	Cathodes		Anodes	
	COFs	Voltage (V)	COFs	Voltage (V)
1	Tb-DANT-COF **[1]**	2.5	TThPP **[2]**	<0.5
2	Tp-DANT-COF **[1]**	2.4	PAT COF **[3]**	<0.5
3	DAAQ-TFP-ECOF **[4]**	2.2	NG-HCP **[5]**	1.4
4	CCP-HATN **[6]**	2.4	COF@CNTs **[7]**	1.5
5	HATN-CMP **[8]**	2.25	CIN-1/CNT **[9]**	1.51

6	PDI-Tc COF **[10]**	2.5	CIN-1/CNT **[11]**	0.8
7	PI-ECOF/rGO **[12]**	2.2	N3-COF **[11]**	0.8
8	NT-COF **[13]**	2.3	PIAQ **[14]**	1.0
9	2DPAI@CNT **[15]**	2.4	Cz-COF-1 and Cz-COF-2 **[16]**	<0.5
10	COF-TRO **[17]**	1.55	IISERP-CON1 **[18]**	0.4
11	BQ1-COF **[19]**	2.64	E-CIN-1/CNT **[3]**	0.4
12	PPTODB-COF **[20]**	2.8	E-SNW-1/CNT **[3]**	0.5
13	E-TP-COF and TP-COF **[21]**	2.25	Tp-Azo-COF **[22]**	<0.2
14	DAPQ-COF **[23]**	2.6	CPOF **[24]**	0.2
15	HATN-AQ-COF **[25]**	2.76	DBA-COF 3 **[26]**	0.5
16	TQBQ-COF **[27]**	1.6	PTT-O@C **[28]**	0.67
17	AZO-1 **[29]**	1.65	COF-1 **[30]**	0.85
18	PCT-1 **[31]**	2.67	PA-COF **[32]**	0.8
19	PIBN-G **[33]**	2.6	TB-COF **[32]**	0.95

Table S2: List of 36 features employed to represent the structural and chemical characteristics of the unit cell of each COF.

Count	Feature Category	Description
8	Composition based	frac_c, frac_h, frac_n, frac_o, frac_(n+o), frac_hal, tdu, te<br><br>frac_X is defined as the fraction of atom type X (X: C, H, N, O, F, Cl, Br)<br><br>frac_X = n_X/N, where n is the number of atom X and N is the total number of atoms in the unit cell<br><br>tdu is the total degree of unsaturation and calculated from $(2(n_C+ 0.5n_N)+ 2 – (n_H, n_F,n_{Cl},n_{Br})))/2$<br>te: electronegative atom count and calculated from $(n_O+n_N+n_F + n_{Cl} + n_{Br})/N$
8	Local connectivity-based	Mean aggregates of the following coordination properties over the rank-ordered redox-sites present in the unit cell. The rank-ordering begins with sp^2 O sites followed by the sp^2 N sites. If certain substructural motifs were present in the unit cell, features were aggregated over all the atoms present in that substructure. Based on their prevalence in DICE, bicarboxylic imides[-C(=O)NC(=O)-], acyl hydrazones [-C(=O)NN] and porphyrin motifs were chosen as those substructures. In the absence of any sp2 O or sp2 N sites, the features were aggregated over all the five membered and six membered rings within the unit cells. Because of the prevalence of the phenyl rings across different families of COFs, the elemental properties (such as the electronegativity, ionization potential) were averaged for a carbon and a hydrogen atom.<br>• number of redox sites<br>• electronegativity<br>• first ionization energy<br>• number of atoms in the first coordination shell<br>• average electronegativity of the first coordination shell<br>• average coordination distance of the coordinated atoms.<br>• average ionization energy of the first coordination shell<br>• average electronegativity of the second coordination shell<br>Except the number of redox-sites, the rest of the seven features are computed as implemented in the PACMOF python library. [34]

5	Local proximity-based	Mean aggregates of the following properties over the proximal heteroatoms (N, O, S)<br>• number of proximal sites<br>• mean electronegativity of the proximal sites<br>• maximum electronegativity of the proximal sites<br>• mean distance of the proximal sites<br>• minimum distance of the proximal sites
10	Electronegativity-weighted radial distribution functions	The spatial correlations of the proximal heteroatoms are calculated using<br><br>**RDF**$^{\chi}\sum_{i,j} \chi_i \chi_j exp[-B(r_{ij} - R)^2]$<br><br>with R ranging from 2 to 6 Å and a B value of 100 $Å^{-1}$ is used.
3	Unit cell based	Three unit cell dimensions along the a, b, c axis
1	Unit cell based	Total number of sp^2 O and sp^2 N sites in the unit cell
1	Unit cell based	Total molecular weight in the unit cell

Section S1: Applicability domain analysis DICE vs. CoRE

To assess whether the CoRE COFs screened using the trained OCV classification model lie within the chemical feature space represented by the DICE COF training set, we performed an applicability domain (AD) analysis based on nearest neighbor (NN) distances in the feature space. The analysis was performed using all 31 features employed to represent the COFs for OCV classification. Prior to distance calculations, the features were standardized using the scaling parameters obtained from the DICE-V COF dataset to prevent differences in feature magnitude from dominating the distance metric. The local density of the training domain was characterized by calculating, for each DICE COF, the Euclidean distance to its nearest neighboring DICE COF in the standardized feature space using the equation below (excluding the self-matches). The 95th percentile of the resulting NN distance distribution was used as the AD threshold,

$$d_{AD} = Q_{0.95}(\{d_i^{DICE}\}),$$

where d_i^{DICE} is the distance between ith DICE COF and its nearest neighboring DICE COF. For each of the remaining CoRE COFs, the Euclidean distance to its nearest DICE COF was then calculated in the same standardized feature space,

$$d_j^{CoRE \to DICE} = \min_i \parallel x_j^{CoRE} - x_i^{DICE} \parallel_2.$$

These remaining CoRE COFs were considered to lie within the applicability domain when its nearest-training-sample distance satisfied

$$d_j^{CoRE \to DICE} \leq d_{AD},$$

whereas structures with distances exceeding this threshold were considered outside the training domain. This analysis therefore evaluates whether a CoRE COF to be screened has a sufficiently close analogue within the feature space represented by the DICE COF training data, rather than relying solely on the classifier prediction probability.

Using this criterion, 814 of 859 (94.7%) of the CoRE COFs (without the DICE-V entries) were found to lie within the AD of the DICE-V training set, while 46 (5.3%) were outside the domain The substantial fraction of CoRE-COFs within the AD indicates considerable overlap between the descriptor spaces represented by the DICE-V and CoRE COF datasets. An inspection of the 46 out-of-domain COFs reveals that these COFs have 1) large unitcell 45-82 Å (a,b lattice), (COF-133, 945, 707, etc.) (2) quite wavy structures leading the large interlayer distance (COF-548, 763), (3) Interpenetrated 2D structure (COF-838), and (4) AA′ antiparallel structures (COF-1074, 52, 1008, 935, etc.)

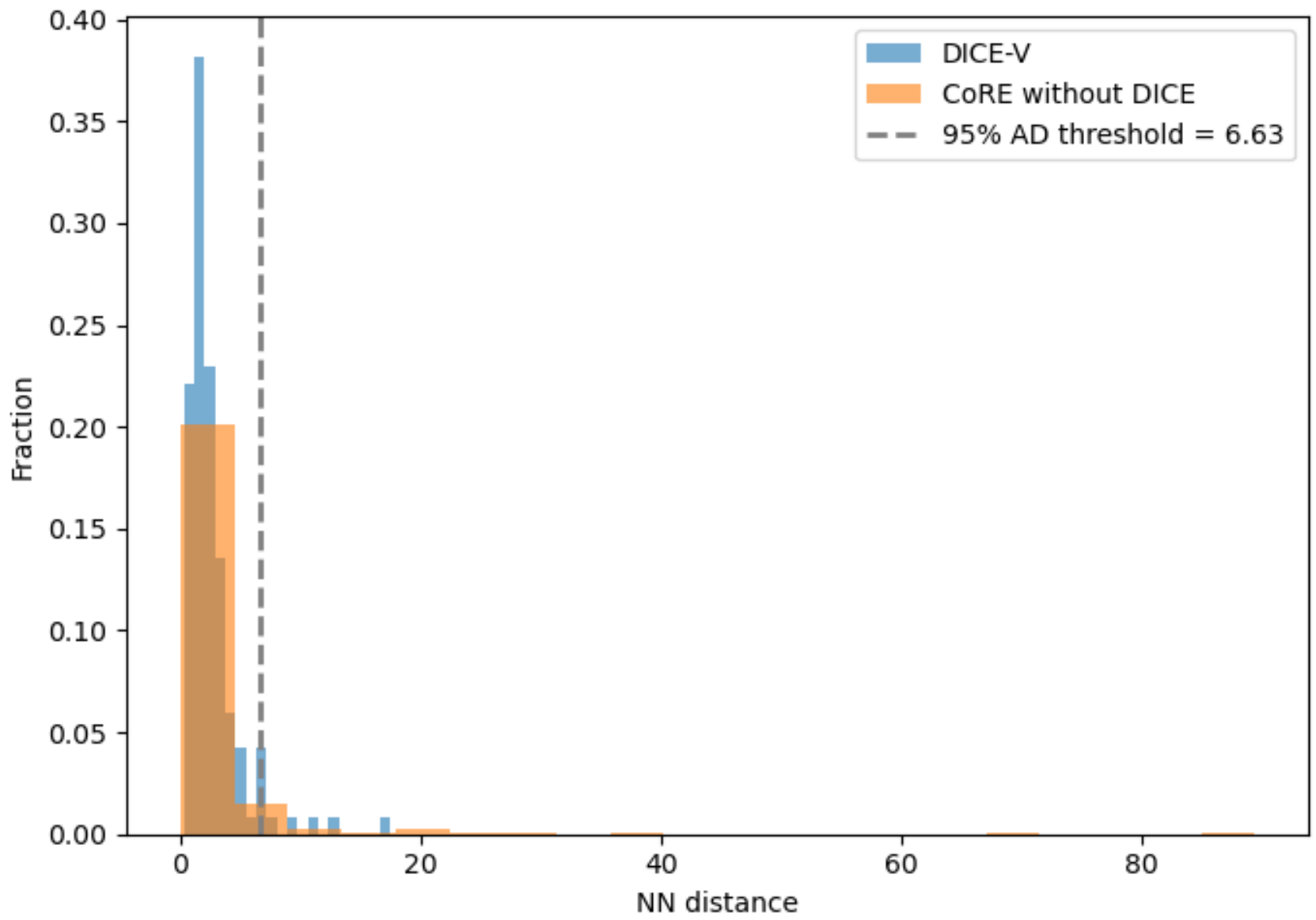

Figure S2: Distribution of the Euclidean distance between each of the rest 859 CoRE COFs and its nearest DICE-V COFs in the standardized descriptor space. The grey dashed line corresponds to the AD threshold, defined as the 95% percentile of the NN distance among the DICE-V COFs.

Table S3: Performance metrics of different ML models for OCV classification task. All models were trained by first using a hyperparameter search by defining a parameter grid and then exploring via *RandomizedSearchCV* with five-fold cross-validation, optimizing the AUC metric.

Model	ROC-AUC	F1-score (weighted)
Random Forest	0.84 ± 0.14	0.85 ± 0.07
kNN	0.81 ± 0.21	0.86 ± 0.07
LR	0.82 ± 0.16	0.82 ± 0.04
SVM	-	0.82 ± 0.04
XGBoost	0.87 ± 0.08	0.85 ± 0.05

Section S2: Construction of the consensus-based XGBoost classifiers for OCV and capacity class prediction

The hard-voting consensus-based XGBoost classification models are trained using the python scripts for OCV and capacity, respectively, provided at https://github.com/dosshubha/COF-ML. The script conducts a workflow that begins by loading the dataset from the *voltage_data.npy or, cap_data_cls.npy* containing the full feature matrix (**X**) and corresponding labels. Class imbalance was addressed by computing a class weight factor (scale_pos_weight = n_negative / n_positive), subsequently used during model training to mitigate bias toward the majority class, which is in this case the AL COFs. First, we conduct an extensive hyperparameter search using the XGBoost algorithm on the complete dataset. A parameter grid was defined and explored via *RandomizedSearchCV* with five-fold cross-validation (CV), optimizing the AUC metric. We optimized the model through a grid search over several hyperparameters, which are given below:

- *n_estimators*: number of gradient boosted trees [100, 200, 300]
- *max_depth*: maximum tree depth for base learners [5, 7, 10]
- *eta*: Boosting learning rate [0.01, 0.1]
- *colsample_bytree*: subsample ratio of columns when constructing each tree [0.25, 0.5],
- *min_child_weight*: minimum sum of instance weight(hessian) needed in a child [1, 10],
- *max_delta_step*: maximum delta step we allow each tree's weight estimation to be [0, 1]

The top five parameter configurations, ranked according to mean cross-validation performance, were selected for subsequent consensus-based model construction. Five XGBClassifier models were instantiated using the optimal hyperparameter sets identified in the previous stage. These base learners were aggregated into a hard-voting ensemble (*scikit-learn VotingClassifier*), designed to leverage the diversity among the individual models and enhance predictive robustness. The ensemble classifier was embedded within a scikit-learn *pipeline*, incorporating a

StandardScaler for feature normalization. Feature selection was performed using Recursive Feature Elimination with Cross-Validation (RFECV), where feature importance scores were averaged across the constituent XGBoost models to identify the most informative predictors.

The complete pipeline was evaluated using 5 × 5 repeated stratified CV, in which five-fold CV was repeated five times with different data partitions, resulting in 25 outer train-test evaluations. Note that, hyperparameter optimization was performed only once on the complete dataset, prior to the outer CV evaluations. For each outer fold, RFECV was performed exclusively on the corresponding training partition using an inner five-fold stratified CV. Balanced accuracy was used as the optimization criterion for feature selection, with a minimum of eight features retained. The number and identity of the selected features were therefore allowed to vary across the outer folds. For each outer fold, the XGBoost ensemble was subsequently trained on the corresponding training partition using the selected features and evaluated on the held-out test partition. Thus, the samples in the outer test partition were not involved in the feature-selection procedure. We measured the performance of the trained classifiers using ROC–AUC, balanced Accuracy, and F1-score. For ROC-AUC, probabilistic outputs from the five constituent XGBoost classifiers were averaged to obtain the consensus class probabilities. Balanced accuracy and weighted F1-score were calculated from the hard-voting class predictions. Given the imbalance between the two classes, we additionally evaluated the precision–recall area under the curve (PR-AUC). Since individual test folds contain only a small number of positive samples, PR-AUC was calculated using pooled out-of-fold (OOF) predictions within each complete five-fold CV repetition. For each repetition, predictions from the five held-out test partitions were pooled such that every COF contributed exactly one prediction obtained when it was excluded from model training. PR-AUC was then calculated from the resulting OOF probabilities over the complete dataset. This procedure resulted in one PR-AUC value for each of the five CV repetitions, from which the mean and standard deviation were calculated.

ROC-AUC measures the ability of the classifier to discriminate between the OCV (anode-like or cathode-like) or capacity classes (> 200 mAh g^{-1} for high capacity and < 200 mAh g^{-1} for low capacity) across all possible decision thresholds, with a value of 1.0 indicating perfect discrimination and 0.5 corresponding to random classification. Balanced accuracy is defined as the average of sensitivity (true positive rate) and specificity (true negative rate), providing a robust measure of performance for imbalanced datasets. Finally, F1-score is the harmonic mean of precision and recall and quantifies the balance between false positives and false negatives.

PR-AUC summarizes the trade-off between precision and recall across classification thresholds and is particularly informative for evaluating minority-class prediction in an imbalanced dataset. For all of these metrics, higher values indicate better classification performance.

We additionally quantified prediction uncertainty with respect to variations in the training data using the standard deviation of the out-of-fold prediction probabilities across the five repeated CV partitions. The median uncertainty estimate for the OCV class prediction across the 137 COFs was 0.029, indicating generally stable predictions across the repeated data partitions.

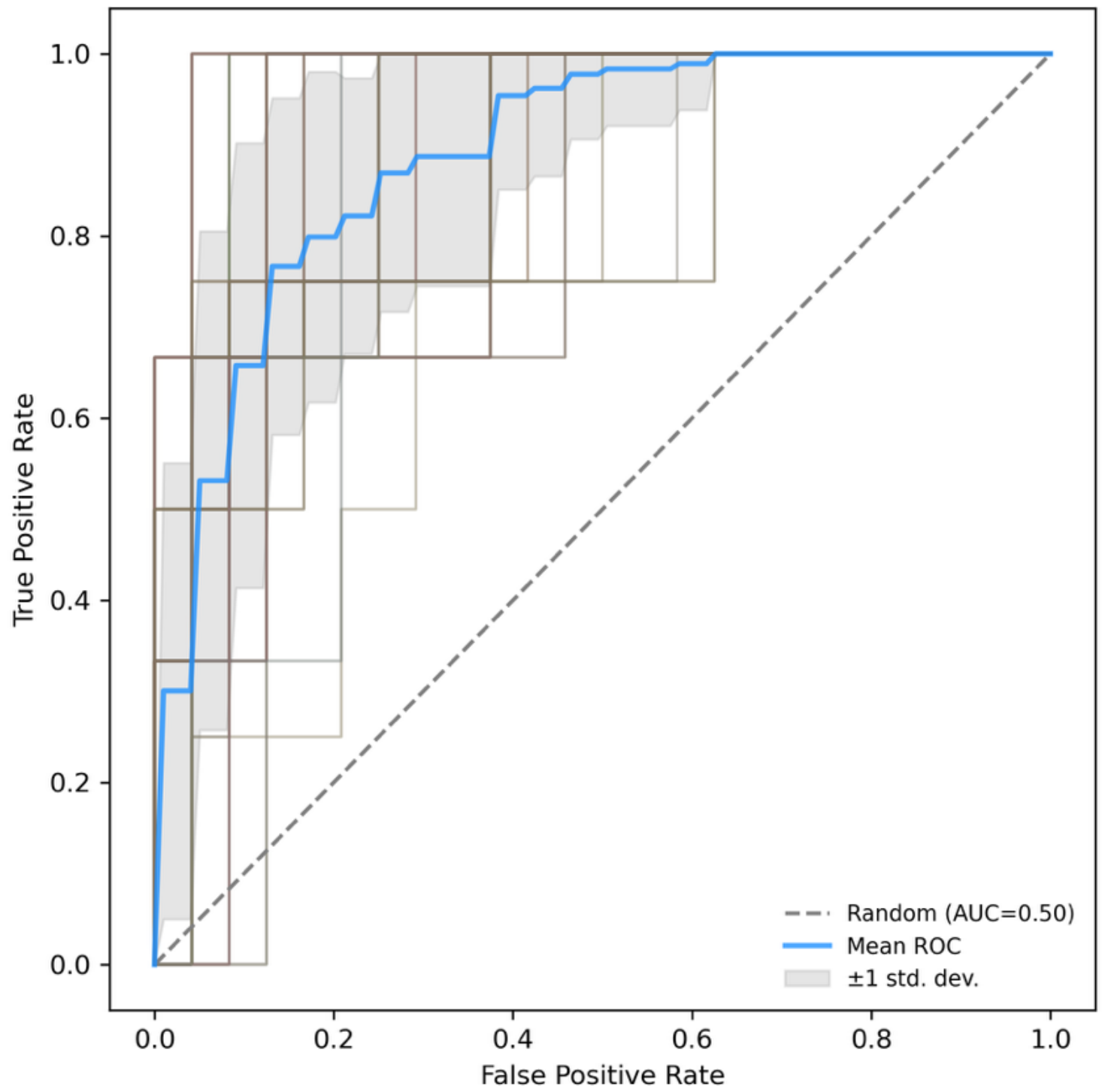

Figure S3: Mean ROC curve describing the performance of the hard-voting consensus-based xGB classification model for voltage class prediction at various threshold values. The ROC curve for 5x5 repeated cross-validation folds are also shown. The grey dashed diagonal line corresponds to the ROC curve expected from a random binary classifier. Various average performance metrics for the 25 outer test folds along with their corresponding standard deviation values are as follows: mean ROC-AUC: 0.88 ± 0.06; mean balanced accuracy: 0.77 ± 0.09 and mean F1-score 0.86 ± 0.05. The out-of-fold PR-AUC is estimated to be 0.51 ± 0.08.

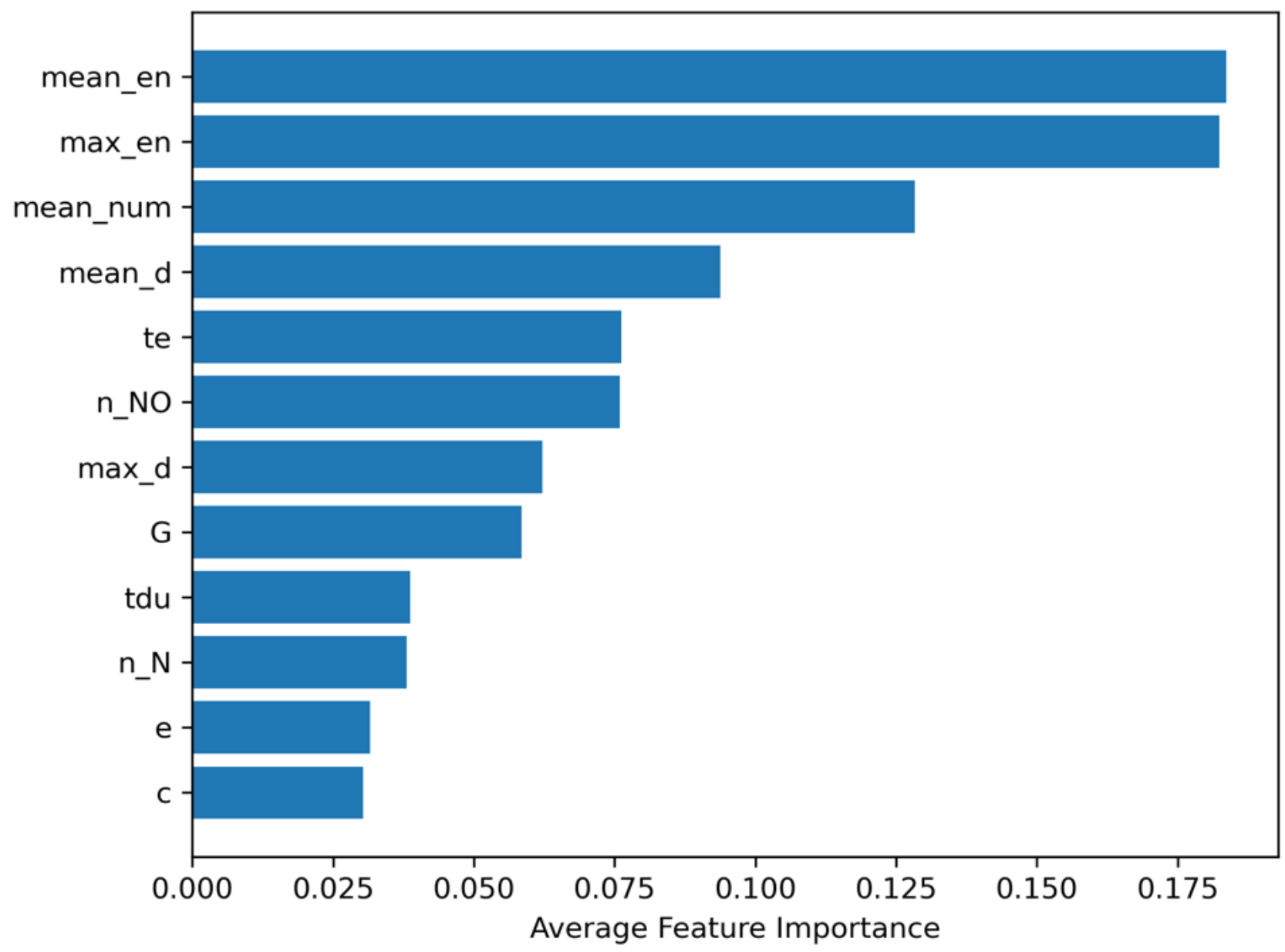

Figure S4: Feature importance analysis of the hard-voting consensus-based xGB classification model for OCV class prediction showing the average importance values for the RFE-reduced feature set.

Section S3: Feature selection stability and grouped permutation importance analysis

To assess the stability of the features identified by RFECV, we evaluated their selection frequency across the 25 outer folds of the 5 × 5 repeated CV. Since RFECV was performed independently within each outer training partition, the selected feature subset was allowed to vary between folds. For each feature, the selection frequency was calculated as the fraction of outer folds in which the feature was retained by RFECV. A high selection frequency therefore indicates that a feature is consistently selected across different partitions of the training data.

To further assess the predictive contribution of the different types of features while reducing the sensitivity of individual feature importance measures to correlated descriptors, we performed a grouped permutation importance analysis. The descriptors were divided into four groups according to their definitions: composition, connectivity, proximal, and electronegativity-weighted radial distribution function features. For capacity classification model the five additional descriptors were included in unit-cell group. However, for each outer fold, all RFECV-selected features belonging to a given group were permuted simultaneously in the corresponding held-out test partition, while the features belonging to the remaining groups were left unchanged. Features within a group were permuted using the same row permutation to preserve their internal relationships. Each permutation was repeated 30 times, and the grouped permutation importance was calculated as the decrease in balanced accuracy (BA) relative to the unpermuted test data,

$$I_g = BA_{baseline} - BA_{permuted,g}$$

where I_g is the permutation importance of feature group g. Thus, a positive value indicates a loss in predictive performance upon disrupting the information encoded by that feature group, whereas values close to zero indicate little change in predictive performance.

The 30 permutation values were first averaged within each outer fold to obtain a single fold-level importance for each feature group, thereby preventing individual folds from receiving unequal statistical weight. The mean and standard deviation of these fold-level values were subsequently calculated across the outer CV folds in which at least one feature from the corresponding group had been selected by RFECV. In addition, 95% confidence intervals (CIs) for the mean grouped permutation importance were estimated by bootstrap resampling of the fold-level importance values using 10,000 bootstrap samples. A feature group was considered to

have a consistently positive contribution when the lower bound of its 95% bootstrap CI was greater than zero.

Table S4: Stability of feature selection for OCV classification across the 25 outer folds. Selection fraction represents the fraction of the 25 outer CV folds in which each feature was selected by the RFECV.

Features	**Times selected**	**Selection fraction**
**n_NO**	25	1.0
**te**	25	1.0
**max_en**	25	1.0
**mean_en**	25	1.0
**mean_num**	25	1.0
**max_d**	23	0.92
**n_H**	23	0.92
**n_N**	20	0.8
**n_C**	19	0.76
**mean_d**	19	0.76
**d**	18	0.72
**n_O**	12	0.48
**g**	12	0.48
**e**	11	0.44
**c**	11	0.44
**tdu**	11	0.44
**b**	10	0.4
**f**	9	0.36
**a**	6	0.24
**G**	5	0.2
**E**	3	0.12
**A**	3	0.12
**h**	1	0.04
**H**	1	0.04
**F**	1	0.04

**D**	1	0.04
**j**	1	0.04
**C**	0	0.0
**B**	0	0.0
**n_halo**	0	0.0
**i**	0	0.0

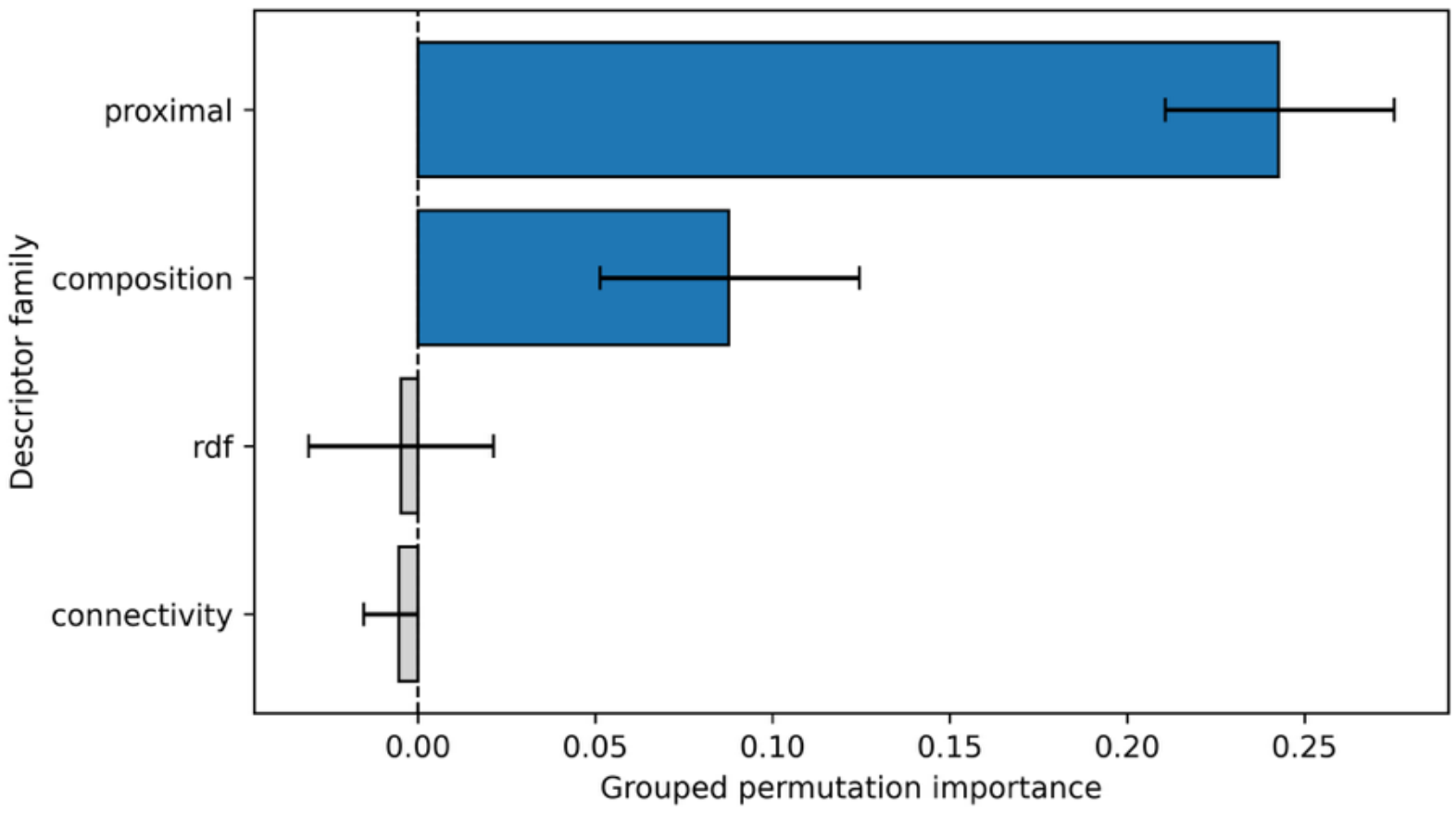

Figure S5: Grouped permutation importance estimated from the decrease in balance accuracy following simultaneous permutation of the RFECV selected features belonging to each group in the held-out outer test set for OCV classification. Values represent the mean +- standard deviations across evaluated outer CV folds. A group was evaluated in an outer fold only when at least one feature belonging to that group was selecetd by RFECV.

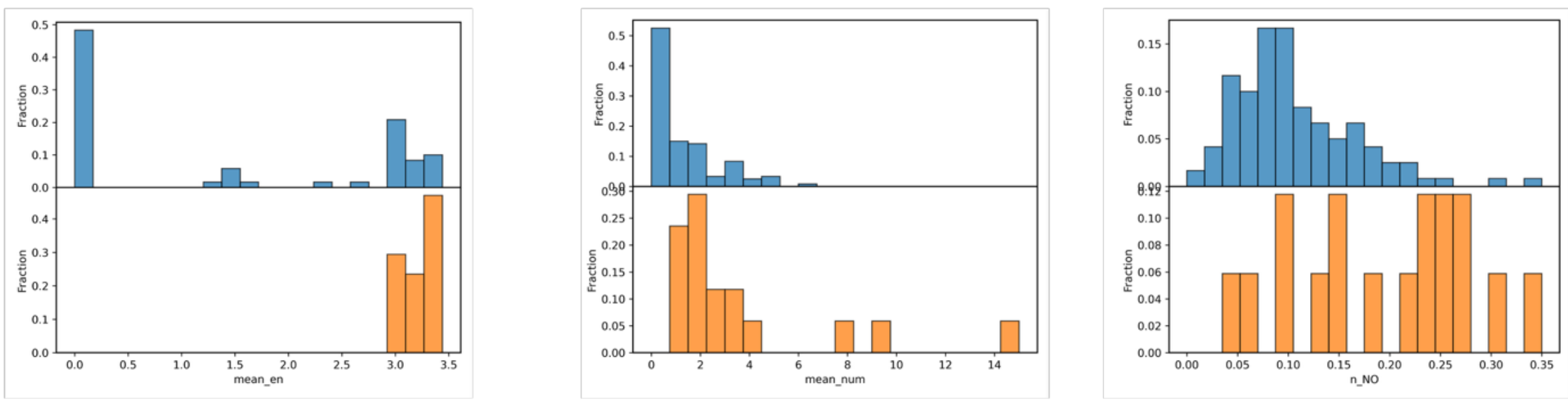

Figure S6: Histogram of feature values for the top RFE-selected features for the anode-like (blue) and the cathode-like (orange) COFs. Counts are normalized by the total number of data points.

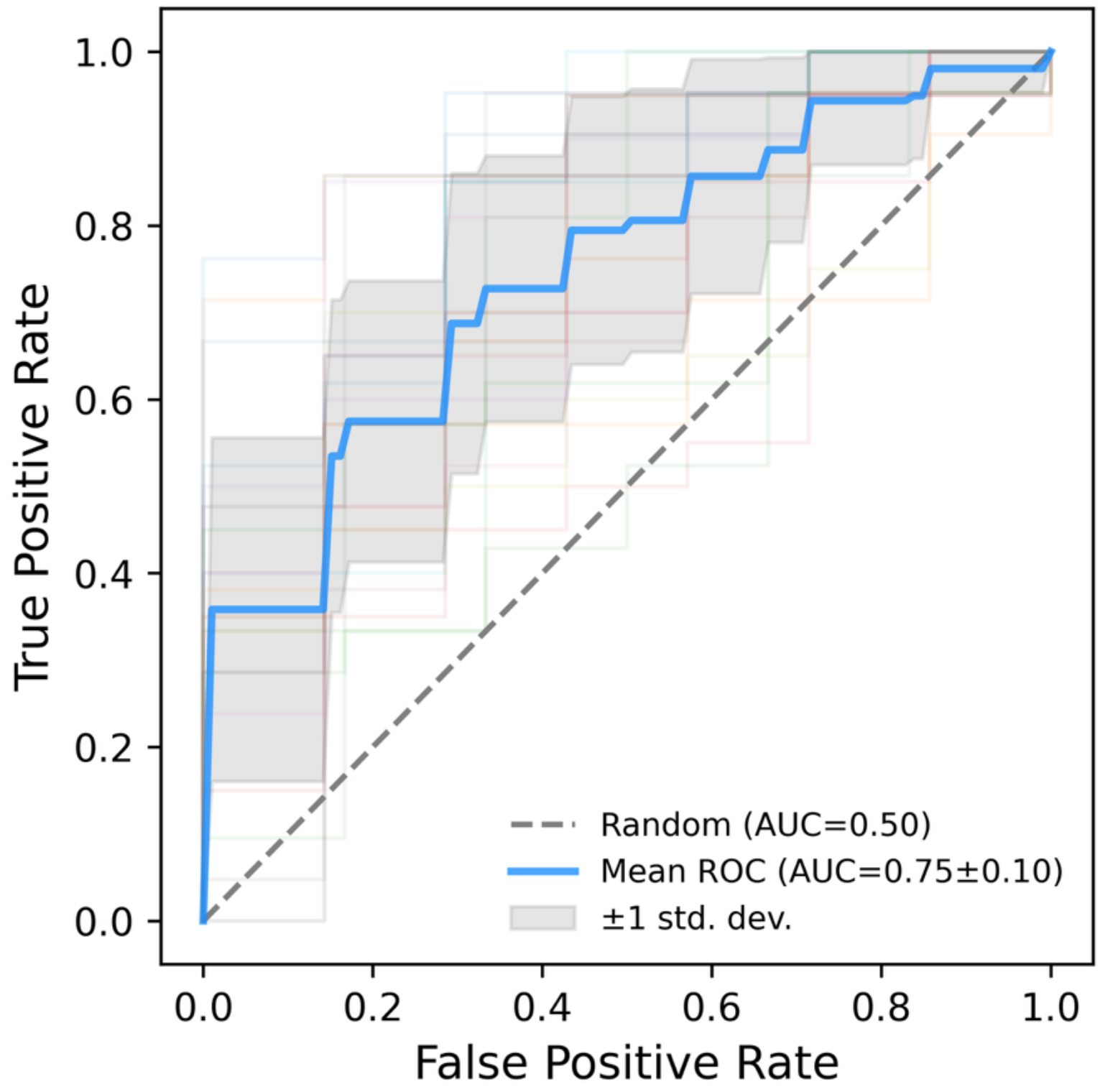

Figure S7: Mean ROC curve describing the performance of the hard-voting consensus-based xGB classification model for capacity class prediction at various threshold values. The ROC Curve for 5x5 repeated cross-validation folds are also shown. The grey dashed diagonal line corresponds to the ROC curve expected from a random binary classifier. Various average performance metrics for the 25 outer test folds along with their corresponding standard deviation values are as follows: mean ROC-AUC: 0.75 ± 0.10; mean balanced accuracy: 0.67 ± 0.10 and F1-score 0.75 ± 0.08.

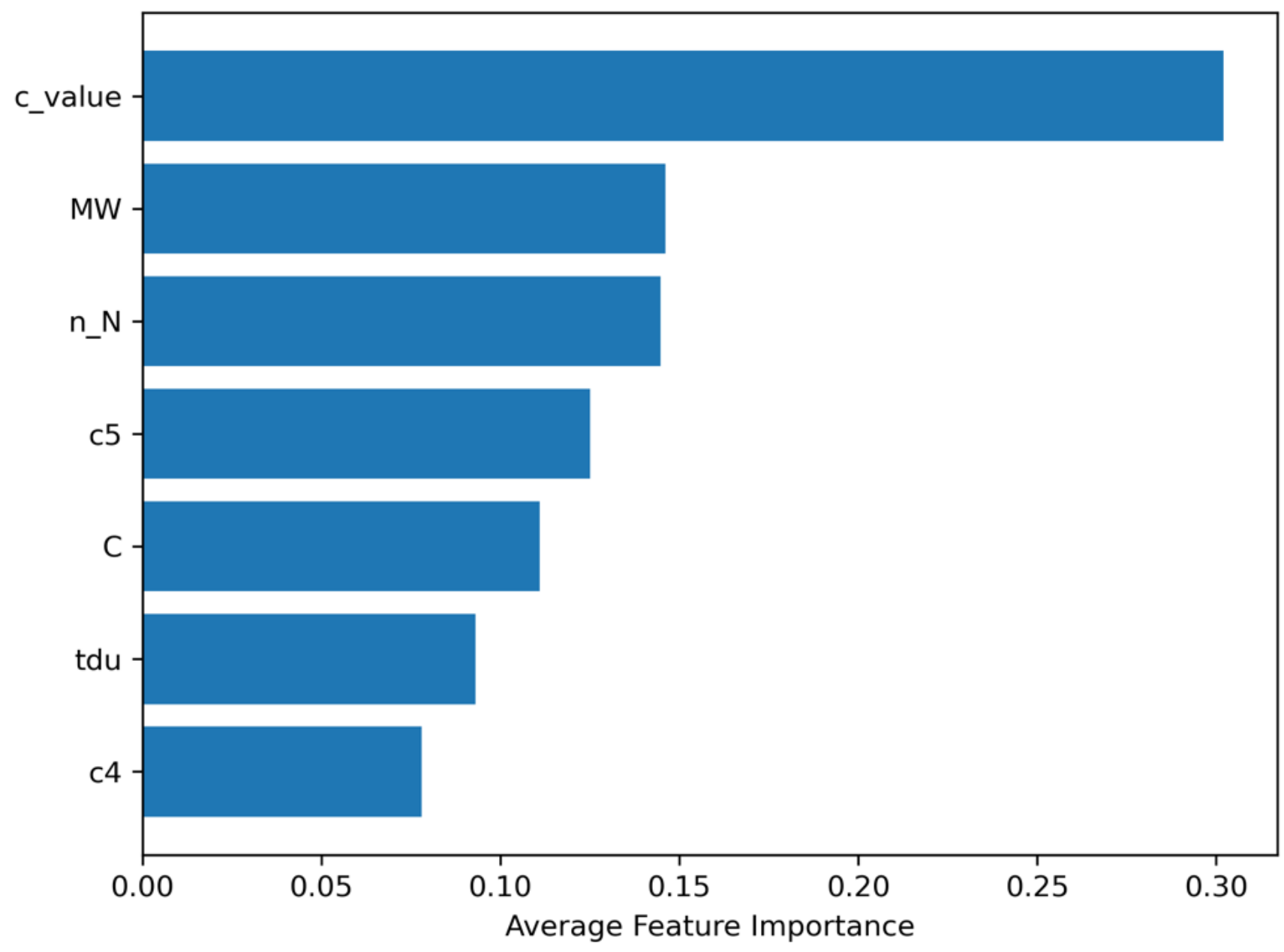

Figure S8: Feature importance analysis of the consensus-based xGBoost classification model for capacity class prediction showing the average importance values for the RFE-reduced feature set.

Table S5: Stability of feature selection for capacity classification across the 25 outer folds. Selection fraction represents the fraction of the 25 outer CV folds in which each feature was selected by the RFECV.

Features	Times selected	Selection fraction
**c_value**	25	1.0
**n_N**	25	1.0
**MW**	25	1.0
**tdu**	19	0.76
**c**	17	0.68
**n_C**	16	0.64
**n_O**	16	0.64
**E**	15	0.6
**nredox**	15	0.6
**a**	15	0.6
**n_NO**	14	0.56

**D**	13	0.52
**a_value**	12	0.48
**d**	12	0.48
**mean_en**	12	0.48
**b_value**	12	0.48
**te**	11	0.44
**mean_num**	10	0.4
**A**	8	0.32
**n_H**	8	0.32
**H**	8	0.32
**max_en**	7	0.28
**mean_d**	7	0.28
**max_d**	7	0.28
**i**	7	0.28
**e**	7	0.28
**b**	7	0.28
**g**	6	0.24
**F**	5	0.2
**f**	5	0.2
**h**	4	0.16
**C**	3	0.12
**j**	3	0.12
**B**	1	0.04
**G**	1	0.04
**n_halo**	1	0.04

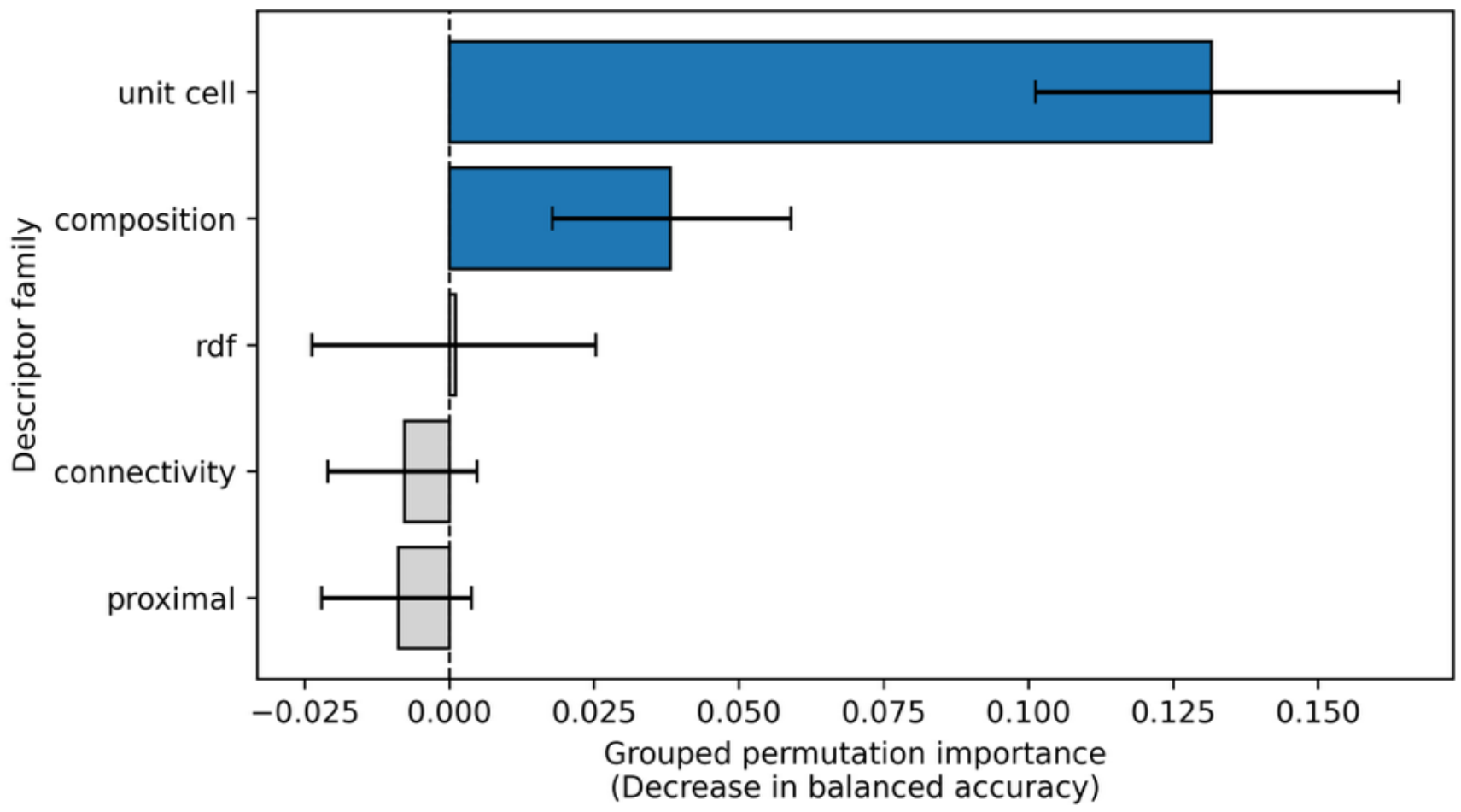

Figure S9: Grouped permutation importance estimated from the decrease in balance accuracy follwing simultaneous permutation of the RFECV selected features belonging to each group in the held-out outer test set for capacity classification. Values represent the mean +- standard deviations across evaluated outer CV folds. A group was evaluated in an outer fold only when at least one feature belonging to that group was selecetd by RFECV.

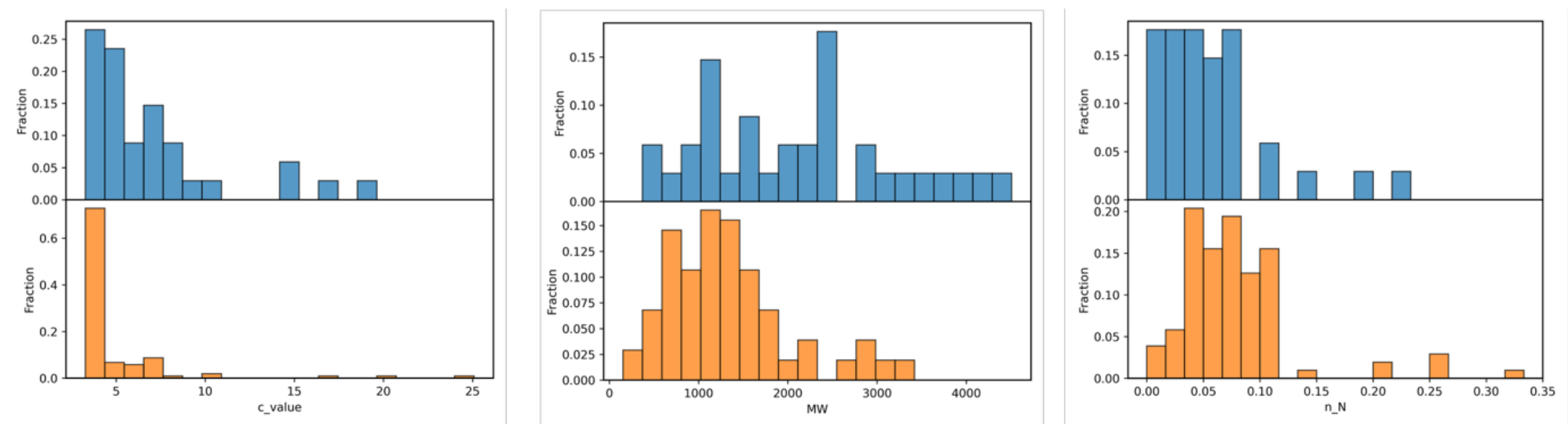

Figure S10: Histogram of feature values for the top RFE-selected features for the COFs with capacity < 200 mAh/g (blue) and > 200 mAh/g (orange) . Counts are normalized by the total number of data points.

Section S4

We have expanded our discussion with a systematic, motif-by-motif comparison between our DFT/ML-derived design rules and independently reported experimental COF electrodes as shown a comprehensive review in Table S6.
Table S6 shows the experimental validation of the derived structure–function design rules from our predictions. The cathode-like behaviour is associated with dense, multidentate N/O pockets, like imide, hexaazatriphenylene cores, β-ketoenamine and acylhydrazone linkages, and carbonyl-rich π-macrocycles. And, the literature holds the similar correlation, as imide COFs (p-DANT-COF, TPDA–PMDA), hexaazatriphenylene COFs (HATN-CMP, HATNPF1-F2), functionalized β-ketoenamine COFs (COF-TRO) and amide COFs (BTH-NT COF) have been reported as cathodes with voltage range of 1.5-4.0 V.
Similarly, the experimental reports (Table X) support the design-rules for anode-like behaviour. Imine COFs (PA-COF/TB-COF, COFs@IM), pyrene-based COFs (PyTTF-COF), and triazole-based COFs (IISERP-CON1) are all predicted as anode with low-voltage window.

Table S6: Motif-by-motif comparison between our DFT/ML-derived design rules and independently reported experimental COF electrodes.

This work		Experimental data	
**Design rules**	**Predicted class**	**Reported COF**	**Reference**
Imide core (dense N/O pocket)	Cathode	p-DANT-COF Cathode: 2.50 V	J. Mater. Chem. A 2016, 4, 18621–18627
Imide core (dense N/O pocket)	Cathode	TPDA–PMDA COF (polyimide, dual N/C=O centers) Cathode: 2–3 V	Angew. Chem. 2022, 134, e202207043.
Hexaazatriphenylene (HAT) core	Cathode	HAQ-COF (HAT-Quinone) , Cathode: 2.93 V	Adv. Mater. 2026 Chem. ACS Sustainable Chem.
Hexaazatriphenylene (HAT) core	Cathode	HATN-CMP: Cathode 1.5-4.0 V	Chem. Commun., 2014, 50, 4788-4790
Hexaazatriphenylene (HAT) core	Cathode	HATNPF1-F2: Cathode	ACS Sustainable Chem. Eng., 2018, 6, 1772–1779.
Functionalized-N-aryl-β-ketoenamine linkage (spatially separated)	Cathode	COF-TRO Cathode: 1.55 V	Angew. Chem. Int. Ed. 2020, 59, 20385 – 20389
amide-linked naphthalimide	Cathode	BTH-NT COF 1.5–3.5 V	Small, 2025, 21, e09595
triazine	Anode	N3-COF Anode: 1.35-1.45 V	J. Mater. Chem. A, 2016, 4, 14106.
Spatially separated triazine redox sites	Anode	PTT-O@C (triazine/EDOT CTF) Anode: 1.0 V	Nano Res., 2022, 15, 9779–9784
Imine linkages	Anode	PA-COF: 0.80 V TB-COF: 0.95 V	Chem. Eur. J., 2019, 25, 15472.
Imine linkages	Anode	TB-COF: 0.95 V	Chem. Eur., J. 2019, 25, 15472.

Pyrene based inter-layer π-rich galleries	Anode	2D PyTTF-COF<br>Anode: 0.6-0.125 V	Angew. Chem. Int. Ed., 2026, 65, e22720
triazole	Anode	IISERP-CON1<br>Anode: 0.02 V	Adv. Energy Mater. 2018, 8, 1702170.
Phenyl and bipyridine	Anode	Alk-Bpy-CMP<br>Anode: 0.5-1.0 V	Small, 2026, 22, e73965
spatially separated imine linkages	Anode	Cz-COFs<br>Anode: 0–0.5 V	Chem. Commun., 2017, 53, 11334–11337.
imine	Anode	COFs@IM<br>Anode: 0.13 V	J. Mater. Chem. A, 2025, 13, 441–449.

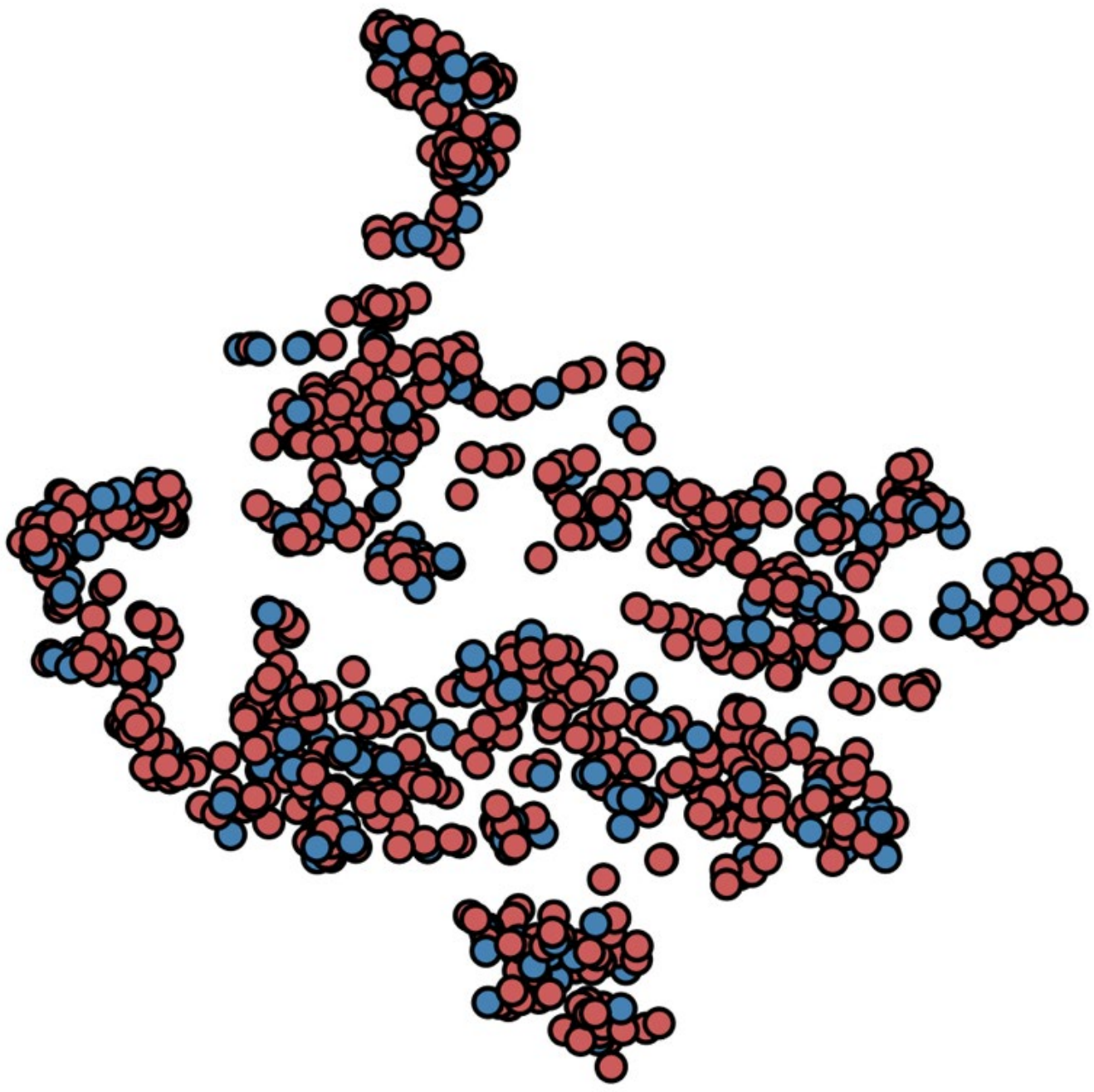

Figure S11: 2D t-distributed stochastic neighbor embedding (t-SNE) map of the CoRE COF database excluding the DICE-C dataset, in which each COF is classified as exhibiting capacity > 200 mAh/g (red) and < 200 mAh/g (blue) based on predictions from the consensus-based hard-voting ensemble of XGBoost classifiers trained on the DICE-C dataset. The embedding was generated using 31 compositional and local descriptors derived from the experimental crystal structures of the CoRE COFs.

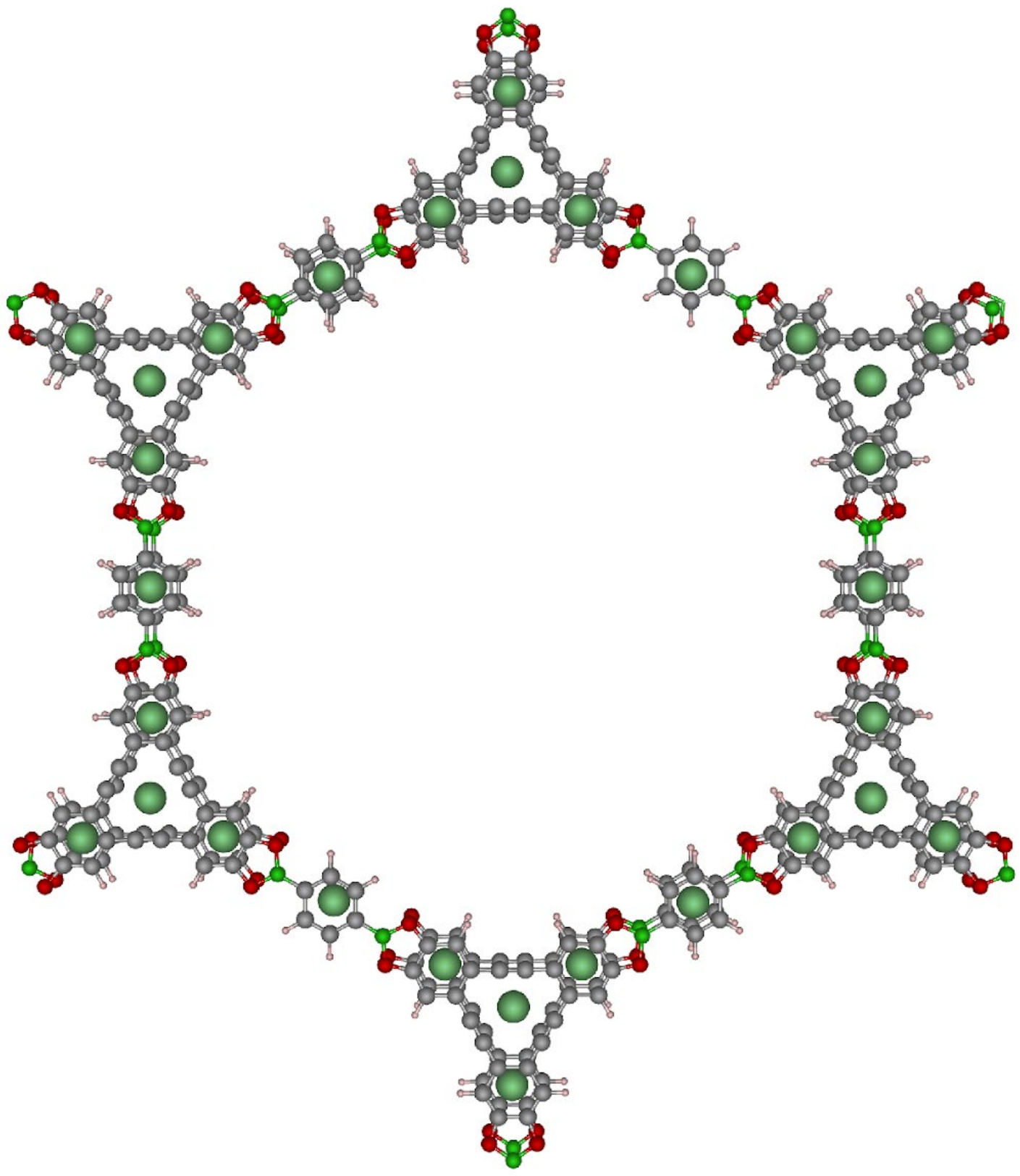

Figure S12: B–O–C linkages are effectively redox-inactive, unlike imines and other RAFs, so Li^+ storage is largely confined to the π-acidic galleries formed between two electron-withdrawing B–O units.

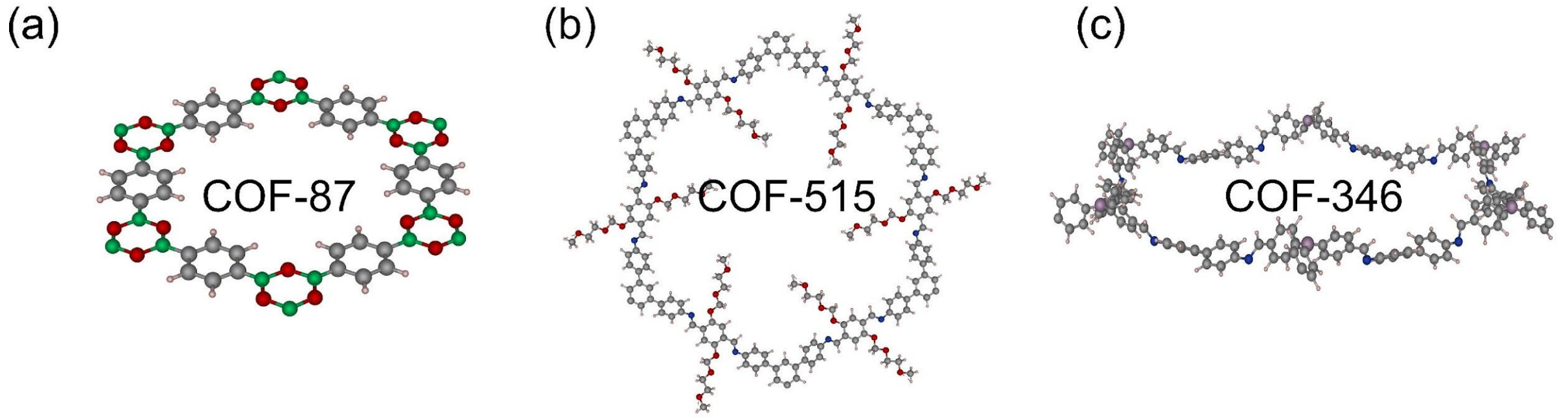

Figure S13: (a) Electrochemically inert boroxine-based COFs in cluster AL1. (b) Example of low-capacity COFs incorporating electron-donating flexible oligomer chains. (c) COF featuring triarylamine units, whose propeller-like phenyl rings do not participate in Li^+ intercalation.

Table S7: List of promising cathode-like COFs from CORE COF database based on the confidence of OCV class prediction. The promising candidates are chosen based on their averaged probability obtained from the hard-voting ensemble exceeding a threshold value of 0.80. The candidates from the DICE COFs, for which explicit DFT-derived OCV values are available, are also included.

SN.	COF Cathode
1	1049
2	1100
3	1103
4	1127
5	1201
6	1223
7	178
8	199
9	233
10	235
11	254
12	328
13	331
14	333
15	334
16	335
17	35
18	353
19	461
20	526
21	538
22	565
23	602
24	635
25	641
26	662
27	664
28	666
29	68
30	793
31	850
32	868
33	908
34	959
35	999
36	153

37	584
38	640
39	1096

Table S8: List of promising anode-like COFs from CORE COF database based on the confidence of OCV class prediction. the promising candidates are chosen based on their averaged probability obtained from the hard-voting ensemble exceeding a threshold value of 0.88. The candidates from the DICE COFs, for which explicit DFT-derived OCV values are available, are also included.

SN.	COF Anode
1	1015
2	1058
3	1059
4	1088
5	1106
6	1121
7	1129
8	1171
9	1203
10	1222
11	134
12	138
13	162
14	168
15	214
16	273
17	274
18	297
19	33
20	338
21	359
22	383
23	419
24	455
25	457
26	55
27	550
28	574
29	582
30	583

31	605
32	627
33	66
34	736
35	815
36	816
37	817
38	819
39	851
40	1133
41	120
42	148
43	156
44	223
45	320
46	366
47	374
48	391
49	492
50	567
51	614
52	626
53	769
54	776
55	84
56	892
57	702
58	899
59	920
60	670
61	524
62	562
63	781
64	658
65	1005
66	1024
67	1067
68	1076
69	1164
70	1199
71	1218
72	1228

73	129
74	150
75	154
76	155
77	167
78	183
79	186
80	2
81	209
82	250
83	263
84	3
85	344
86	384
87	4
88	421
89	43
90	452
91	458
92	468
93	488
94	554
95	575
96	596
97	613
98	63
99	634
100	647
101	696
102	740
103	743
104	752
105	814
106	825
107	986
108	1033
109	285
110	38
111	537
112	61
113	645
114	810

115	848
116	927
117	934
118	955
119	936
120	867
121	31
122	53
123	434
124	463
125	869

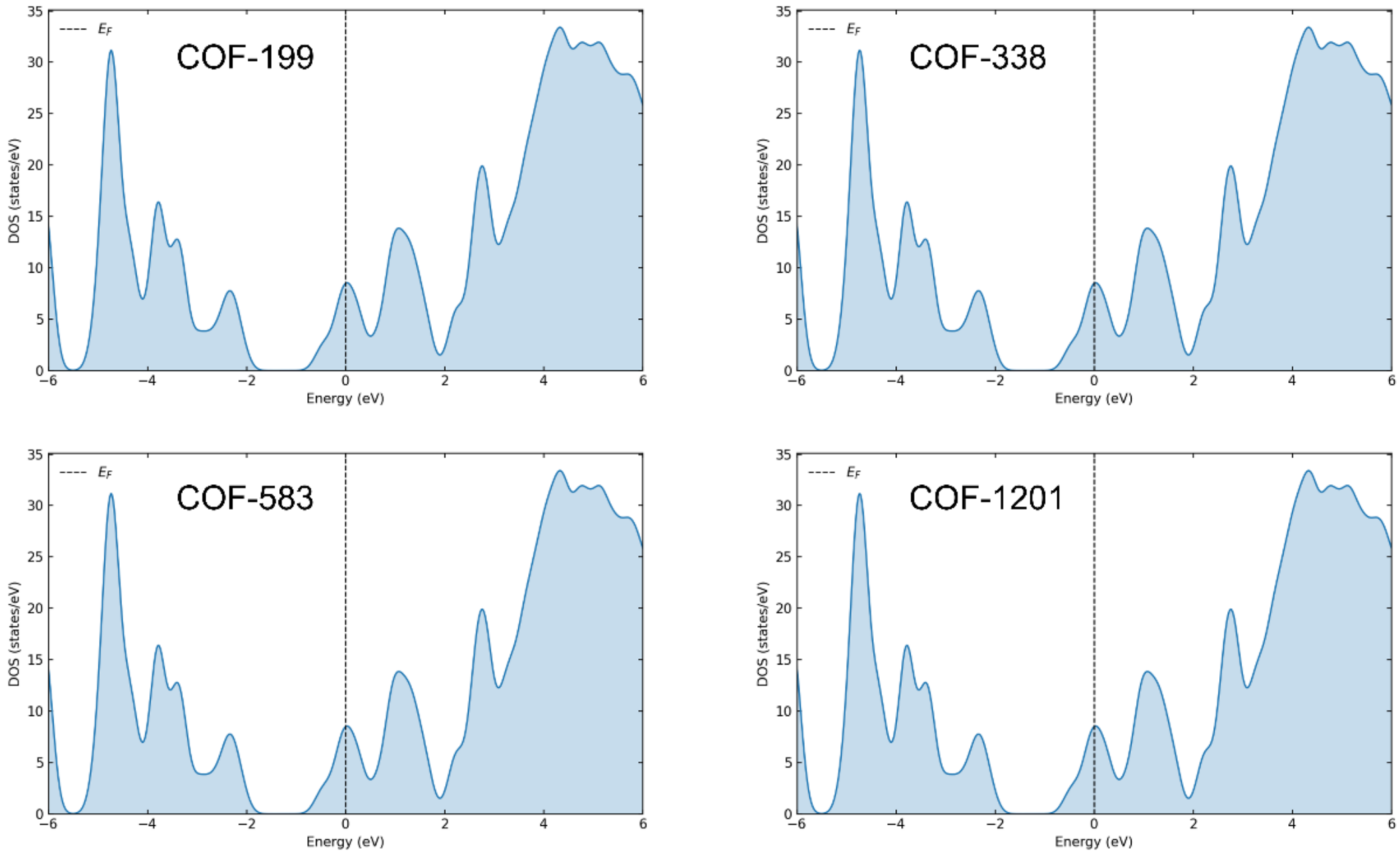

Figure S14: Density of states (DOS) of the some selected lead COFs in their Li inserted first stage. Red dotted lines indicate the Fermi level.

Table S9: Electronic conductivity screening of 31 screened candidates.

COFs	Electronic nature
1201	Metallic
199	
335	
461	
538	
662	
664	
666	
68	
868	
959	
178	
254	
908	
134	
162	
582	
383	
583	
338	
55	

129<br>186<br>634<br>740<br>955<br>936<br>53	
614	0.84 eV
810	0.35 eV
1088	0.18 eV

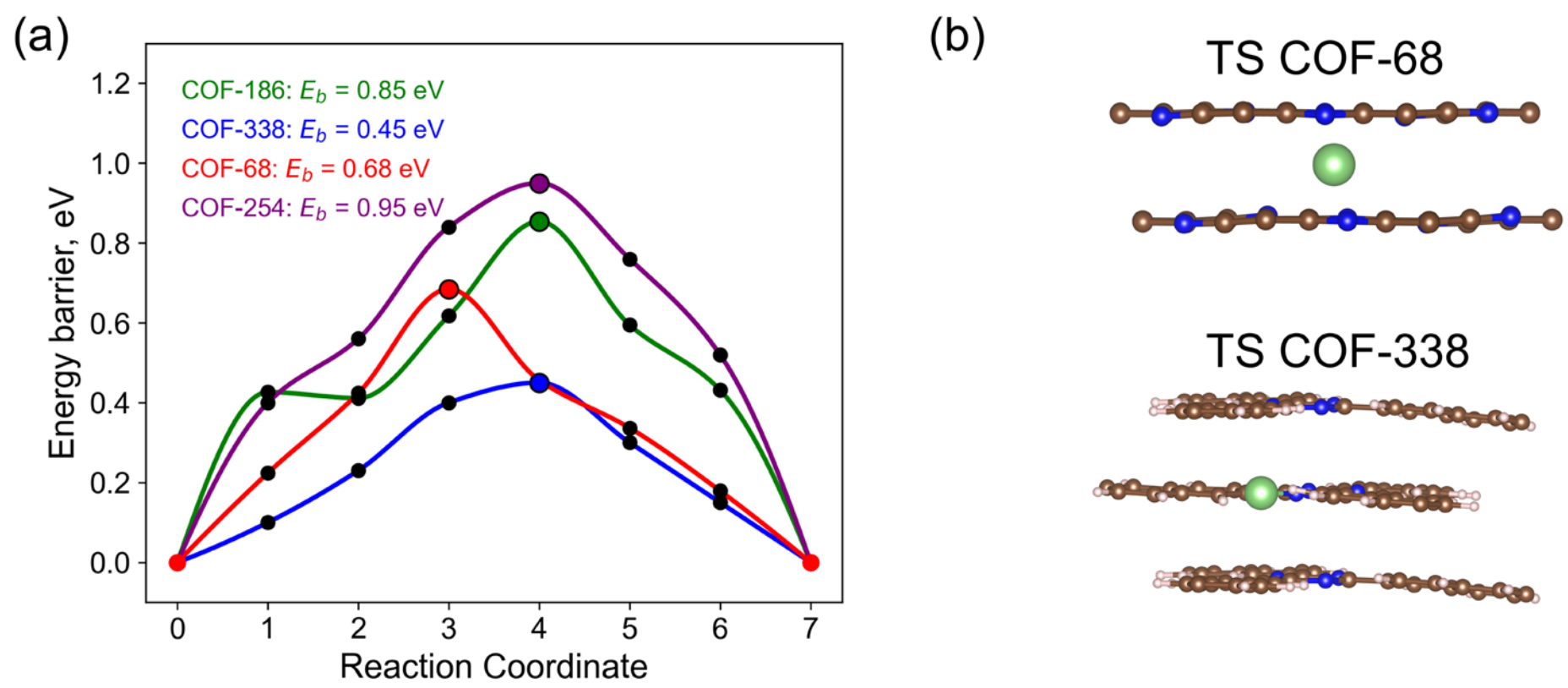

Figure S15: (a) Li^+ diffusion barrier of four representative COFs. (b) Transition state (TS) structures of COF-68 and COF-338. Here, brown, light pink, blue and green balls denote elements C, H, N and Li, respectively.

A full CI-NEB (climbing-image nudged elastic band) treatment of all 28 lead candidates to get the Li^+ diffusion barriers is not computationally feasible within the scope of this study. We have therefore selected four representative COFs spanning the voltage range in our study, two anode (COF-186, 0.26 V and COF-338, 0.62 V) and two cathode (COF-68, 2.30 V and COF-254, 2.71 V) to get the approximate Li^+ diffusion barrier range for the two categories. As shown in Figure S15, for each COF, we identified the lowest-energy Li^+ migration pathway between adjacent stable adsorption sites. The diffusion barrier for COF-186, COF-338, COF-68 and COF-254 are 0.85 eV, 0.45 eV, 0.68 eV, and 0.95 eV, respectively. We did not find any correlation between the voltage/binding energy and Li^+ diffusion barrier in these COFs, as the barrier is governed by the energy of transition state (TS) structures possible for different COFs (Figure S15b). However, we find that the barriers fall within the typical range 0.2-1.7 eV reported for Li^+ diffusion barrier in COFs with varying pore size and chemistry.[35-37]

Table S10: List of lead cathode and anode COFs following a voltage-based filtering and metallic-character screening.

SN.	Cathode		Anode	
	**COF-ID**	Voltage	**COF-ID**	Voltage
1	1201	2.24	134	0.38
2	199	2.65	162	0.43
3	335	2.36	582	0.52
4	461	2.00	383	0.59
5	538	2.29	583	0.38
6	662	2.37	338	0.62
7	664	2.38	55	0.62
8	666	2.93	129	0.44
9	68	2.30	186	0.26
10	868	2.04	634	0.53
11	959	2.69	740	0.44
12	178	2.20	955	0.42
13	254	2.71	936	0.47
14	908	2.00	53	0.54

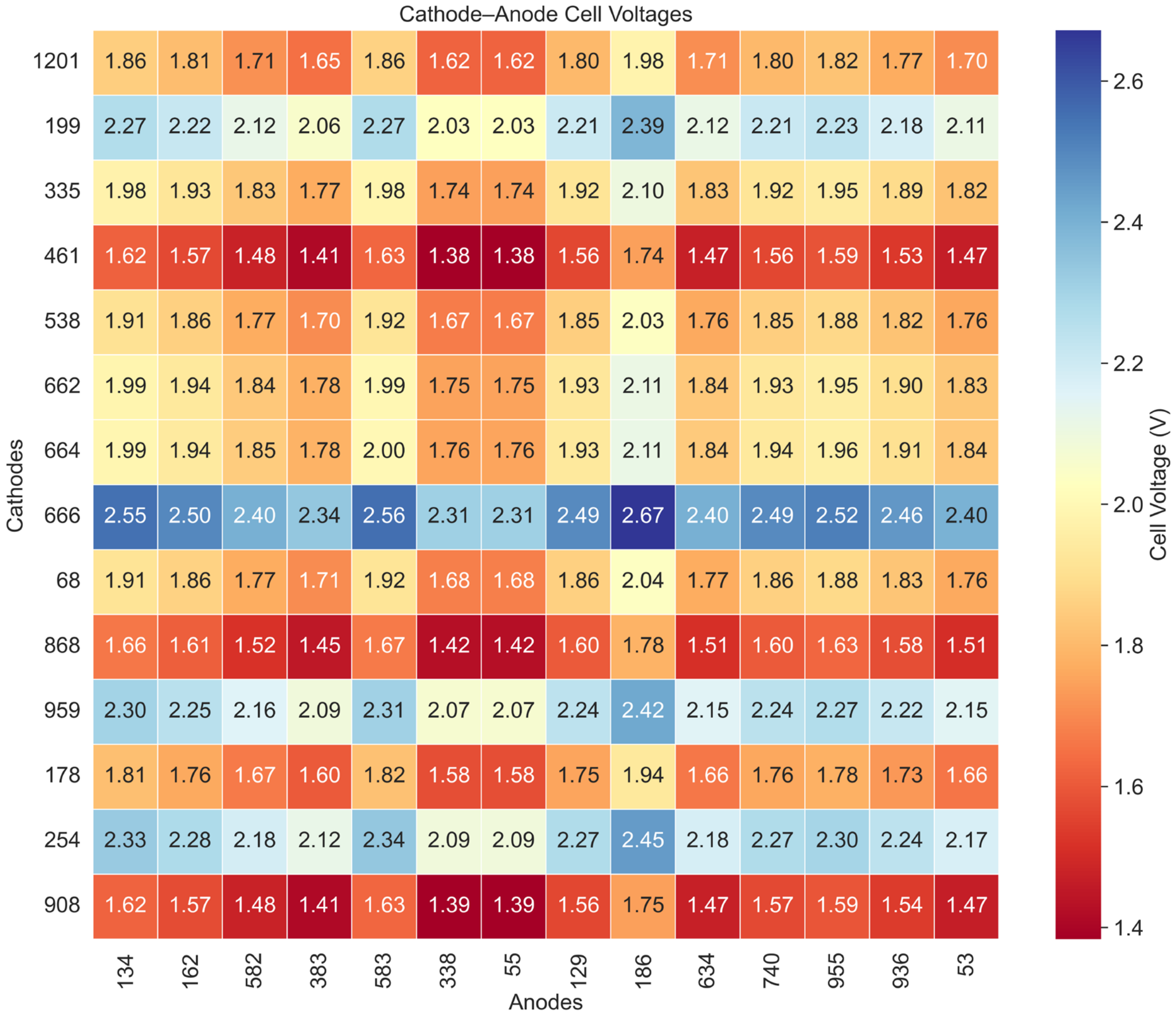

Figure S16: Heatmap showing the top-performing anode–cathode COF combinations for all-organic lithium batteries (AOLBs) with full-cell voltages.

Table S11: Top-performing anode–cathode COF combinations for all-organic lithium batteries (AOLBs) with full-cell voltages, full cell capacities and energy densities.

No.	Cathode ID	Anode ID	Cell Voltage (V)	Cell Capacity (mAh/g)	Cell energy density (Wh/kg)
1	199	186	2.3894	250.39	598.29
2	68	186	2.0378	290.93	592.87
3	666	186	2.6721	215.2	575.04
4	662	186	2.1088	253.15	533.83
5	199	583	2.2733	223.6	508.32
6	666	583	2.556	195.11	498.7
7	664	186	2.1149	229.95	486.32
8	199	582	2.1222	223.6	474.53
9	666	582	2.4049	195.11	469.22
10	959	186	2.4241	184.35	446.88
11	666	936	2.4631	171.04	421.29
12	199	936	2.1804	192.55	419.84
13	666	134	2.5488	162.72	414.73
14	199	134	2.2661	182.07	412.57
15	538	186	2.0339	202.63	412.12
16	666	129	2.4912	161.1	401.32
17	199	129	2.2085	180.04	397.62
18	335	186	2.1004	186.23	391.17
19	959	583	2.308	169.4	390.99
20	254	186	2.4512	152.82	374.59
21	666	740	2.4926	148.95	371.29
22	666	53	2.3956	154.68	370.54
23	666	634	2.4005	154.26	370.29
24	959	582	2.157	169.4	365.4
25	199	740	2.2099	165.01	364.65
26	199	53	2.1129	172.06	363.54
27	199	634	2.1178	171.54	363.28
28	666	955	2.517	143.66	361.59
29	199	955	2.2343	158.53	354.21
30	666	162	2.4987	137.12	342.62
31	666	338	2.313	145.98	337.65
32	959	936	2.2152	150.96	334.4
33	199	162	2.216	150.6	333.74
34	254	583	2.3351	142.41	332.54
35	959	134	2.3008	144.44	332.33
36	199	338	2.0303	161.36	327.61
37	959	129	2.2432	143.16	321.14
38	254	582	2.1841	142.41	311.02
39	959	740	2.2447	133.49	299.64
40	666	383	2.3398	126.78	296.65
41	959	53	2.1476	138.07	296.52

42	959	634	2.1526	137.73	296.47
43	959	955	2.269	129.22	293.21
44	666	55	2.313	126.0	291.43
45	254	936	2.2423	129.14	289.57
46	254	134	2.3279	124.34	289.45
47	199	383	2.0571	138.23	284.35
48	254	129	2.2703	123.39	280.14
49	959	162	2.2508	123.9	278.88
50	199	55	2.0303	137.29	278.75
51	959	338	2.0651	131.09	270.72
52	254	740	2.2718	116.14	263.84
53	254	634	2.1796	119.34	260.11
54	254	53	2.1747	119.59	260.07
55	254	955	2.2961	112.9	259.22
56	254	162	2.2779	108.82	247.87
57	959	383	2.0919	115.4	241.41
58	254	338	2.0922	114.32	239.18
59	959	55	2.0651	114.75	236.97
60	254	383	2.1189	102.2	216.57
61	254	55	2.0922	101.69	212.76

Section S5: Computational methodology

Validation of the voltage and capacity equations for COFs

The calculated OCV and capacity values were validated by comparison with experimentally reported data for selected COF systems. Table S12 presents both the experimental and theoretical values, calculated using equations 3 and 4 (main manuscript). Additionally, Table S2 includes the optimized structures of COFs with incorporated Li ions that were used for the theoretical calculations. This comprehensive comparison validates our computational methodology and provides a reliable dataset for training our ML classifiers.

Table S12: Table showing the studied COF system, theoretical (Theory) and experimental (Exp.) voltage and capacities and the corresponding structures to these values.

COF	Voltage (V)		Capacity (mAh/g)	
	Theory	Exp.	Theory	Exp.
COF-233	2.1-2.3	2.4 [38]	82.5	75 (85%) [38]
COF-461	-	-	145.5	144.4[39]
COF-662	2.3-2.4	2.4-2.5[40]	-	-
COF-TRO	1.5	1.55[41]	274	268[41]

The reliability of our computational protocol is validated by benchmarking the calculated OCV and theoretical capacity against experimentally reported voltages and/or capacities for COF-233 (DTP-ANDI-COF), COF-461 (Tb-DANT-COF), and COF-662 (Hex-Aza-COF-1) from the CoRE-COF database. In addition, we also consider the well-studied COF-TRO for which experimental data is available. All resulting voltages and capacities are reported in Table S2. The theoretical and experimental results are in excellent agreement (within 4% as the maximum deviation): COF-662 exhibits the highest voltage of 2.3–2.4 V (experimental: 2.4–2.5 V), while COF-TRO shows the lowest voltage of 1.5 V (experimental: 1.55 V) and a theoretical capacity of 274 mAh/g (experimental: 268 mAh/g).

Section S6: Sensitivity to the classification thresholds.

To assess the sensitivity of the classification results to the OCV threshold used to define anode- and cathode-like COFs, the model-training procedure was repeated using thresholds of 1.5, 1.6, and 1.7 V. As shown by Table S13, the predictive performance was only moderately affected by these changes, with the ROC–AUC varying by at most 0.06 relative to the model based on the original 1.6 V threshold. Thus, while the overall predictive performance is relatively insensitive to moderate variations in the classification threshold, increasing the OCV boundary leads, as expected, to a more restrictive identification of cathode-like COFs (e.g. 15% for 1.5 V to 7% for 1.7 V). A similar analysis was performed with the capacity classification model by retraining and evaluating the model using thresholds of 150 and 250 mAh g^{-1}. The balanced accuracy ranged from 0.69 to 0.62, while the ROC–AUC spans from 0.81 to 0.68. These results indicate that the overall classification performance is reasonably robust to moderate variation in the capacity threshold.

Table S13: Performance of the OCV and capacity classification models with various decision thresholds.

**Decision Threshold**	**ROC-AUC**	**Balanced accuracy**
1.5 V	0.86±0.07	0.69±0.13
1.6 V	0.88±0.06	0.77±0.09
1.7 V	0.82±0.09	0.63±0.12
150 mAh/g	0.81±0.08	0.69±0.11
200 mAh/g	0.75±0.10	0.67±0.10
250 mAh/g	0.68±0.10	0.62±0.08

Table S14: Performance comparison of the OCV and capacity classification models when the features are extracted from the unrelaxed or relaxed CIFs of the COFs. For the OCV classifier, the predictive performance remained essentially unchanged, whereas the capacity classifier showed only a modest improvement upon structural relaxation (ROC-AUC: 0.75 for unrelaxed vs. 0.78 for relaxed structures). Importantly, the main conclusions from the feature-importance analysis were preserved. For the OCV classifier, local and compositional features contributed approximately 78% (77%) and 22% (23%), respectively, for relaxed (unrelaxed) structures. Within the local features, relaxation resulted in some redistribution of importance: the contribution of proximal descriptors decreased from 65% to 51%, accompanied by increased contributions from RDF (6% to 9%) and connectivity features (6% to 18%). Overall, these results indicate that both the predictive performance and the principal feature-importance trends are relatively insensitive to whether features are extracted from relaxed or unrelaxed structures.

We therefore retained the models based on unrelaxed experimental structures for screening the remaining CoRE COF dataset. This avoids the additional computational cost of geometry optimization and, importantly, enables newly reported experimental structures to be screened directly from their CIFs.

**Property**	**ROC-AUC**	**Balanced accuracy**
**OCV (unrelaxed)**	**0.88**	**0.77**
**OCV (relaxed)**	**0.88**	**0.76**
**Capacity (unrelaxed)**	**0.75**	**0.67**
**Capacity (relaxed)**	**0.78**	**0.70**

Table S15: As an additional validation of the OCV classifier, we randomly selected eight COFs from the CoRE COF (without the DICE entries) dataset without consideration of their ML-predicted classes. Their OCV values were calculated explicitly using the same DFT protocol employed for the labelling of the DICE COFs. All eight structures were classified as anode-like based on their computed OCV values, in agreement with the ML predictions. We note that the randomly selected subset contained no DFT-validated cathode-like structures. Given the predominance of the anode-like COFs in the dataset (around 85%), the absence of cathode-like structures in this randomly drawn subset is not unexpected. Consequently, this blind test provides additional validation of the anode-like predictions but does not independently assess cathode-like classes.

COF ID	Voltage Calculated with DFT (V)	OCV class prediction with ML
30	0.72	Anode
472	1.39	Anode
165	0.88	Anode
213	1.00	Anode
330	1.03	Anode
772	1.22	Anode
827	1.01	Anode
972	1.33	Anode

References

1. DOI: 10.1002/aenm.202100172.
2. DOI: 10.1021/acsami.5b12370.

3. Kang, H.; Liu, H.; Li, C.; Sun, L.; Zhang, C.; Gao, H.; Yin, J.; Yang, B.; You, Y.; Jiang, K. C.; Long, H.; Xin, S. *ACS Appl. Mater. Interfaces* **2018**, *10*, 37023–37030.
4. *J. Am. Chem. Soc.* **2017**, *139*, 4258–4261.
5. DOI: 10.1016/j.nanoen.2017.08.038.
6. DOI: 10.1002/ange.201812685.
7. *Nat. Commun.* **2018**, *9*, 576.
8. DOI: 10.1039/D4TA02085E.
9. *J. Mater. Chem. A* **2025**, *13*, 10337–10357.
10. *ACS Appl. Mater. Interfaces* **2017**, *9*, 15631.
11. DOI: 10.1002/pol.20210940.
12. DOI: 10.1039/C9NR00088G.
13. DOI: 10.1002/ange.201806596.
14. *J. Mater. Chem. A* **2019**, *7*, 2368.
15. *Adv. Mater.* **2019**, *31*, 1901478.
16. *Chem. Commun.* **2017**, *53*, 11334–11337.
17. *Angew. Chem., Int. Ed.* **2020**, *59*, 20385.
18. *Adv. Energy Mater.* **2018**, *8*, 1702170.
19. *Nano Energy* **2020**, *70*, 104498.
20. DOI: 10.1002/cssc.201903007.
21. *Adv. Funct. Mater.* **2021**, 2101019.
22. *ACS Energy Lett.* **2020**, *5*, 1022–1031.
23. DOI: 10.1002/aenm.202101880.
24. Yu, X.; Li, C.; Ma, Y.; Li, D.; Li, H.; Guan, X.; Yan, Y.; Valtchev, V.; Qiu, S.; Fang, Q. *Microporous Mesoporous Mater.* **2020**, *299*, 110105.
25. DOI: 10.1002/anie.202207043.
26. DOI: 10.1039/C9ME00104B.
27. Shi, R.; Liu, L.; Lu, Y.; et al. Nitrogen-Rich Covalent Organic Frameworks with Multiple Carbonyls for High-Performance Sodium Batteries. *Nat. Commun.* **2020**, *11*, 178.
28. *Polymers* **2021**, *13* (19), 3300. DOI: 10.3390/polym13193300.
29. Singh, V.; Kim, J.; Kang, B.; Moon, J.; Kim, S.; Kim, W. Y.; Byon, H. R. Thiazole-Linked Covalent Organic Framework Promoting Fast Two-Electron Transfer for Lithium-Organic Batteries. *Adv. Energy Mater.* **2021**, *11*, 2003735.
30. DOI: 10.1016/j.physb.2023.415027.
31. DOI: 10.1039/C9SC04175C.
32. Chen, H.; Zhang, Y.; Xu, C.; Cao, M.; Dou, H.; Zhang, X. *Chem. Eur. J.* **2019**, *25*, 15472.
33. *Angew. Chem., Int. Ed.* **2018**, *57*, 9443.
34. J. Chem. Theory Comput. 2021, 17, 5, 3052–3064.
35. *J. Phys. Chem. Lett.* 2026, 17, 5156−5163.
36. *Nano-Micro Lett.* 2026, *18*, 163.
37. *J. Phys. Chem. C* 2019, *123*, 30155−30164
38. https://www.nature.com/articles/srep08225
39. https://pubs.rsc.org/en/content/articlehtml/2016/ta/c6ta07606h
40. https://www.cell.com/action/showPdf?pii=S2451-9294%2820%2930035-8
41. Angew. Chem. Int. Ed. 2020, **59**, 20385–20389.
